# RNA m^5^C oxidation by TET2 regulates chromatin state and leukaemogenesis

**DOI:** 10.1038/s41586-024-07969-x

**Published:** 2024-10-02

**Authors:** Zhongyu Zou, Xiaoyang Dou, Ying Li, Zijie Zhang, Juan Wang, Boyang Gao, Yu Xiao, Yiding Wang, Lijie Zhao, Chenxi Sun, Qinzhe Liu, Xianbin Yu, Hao Wang, Juyeong Hong, Qing Dai, Feng-Chun Yang, Mingjiang Xu, Chuan He

**Affiliations:** 1 Department of Chemistry, The University of Chicago, Chicago, IL USA; 2 Howard Hughes Medical Institute, The University of Chicago, Chicago, IL USA; 3 Department of Molecular Medicine, University of Texas Health Science Center at San Antonio, San Antonio, TX USA; 4 Department of Biochemistry and Molecular Biology and Institute for Biophysical Dynamics, The University of Chicago, Chicago, IL USA; 5 Department of Cell Systems and Anatomy, University of Texas Health Science Center at San Antonio, San Antonio, TX USA; 6 May’s Cancer Center, University of Texas Health Science Center at San Antonio, San Antonio, TX USA

**Keywords:** Molecular biology, Chromatin

## Abstract

Mutation of tet methylcytosine dioxygenase 2 (encoded by *TET2*) drives myeloid malignancy initiation and progression^1–3^. TET2 deficiency is known to cause a globally opened chromatin state and activation of genes contributing to aberrant haematopoietic stem cell self-renewal^4,5^. However, the open chromatin observed in TET2-deficient mouse embryonic stem cells, leukaemic cells and haematopoietic stem and progenitor cells^5^ is inconsistent with the designated role of DNA 5-methylcytosine oxidation of TET2. Here we show that chromatin-associated retrotransposon RNA 5-methylcytosine (m^5^C) can be recognized by the methyl-CpG-binding-domain protein MBD6, which guides deubiquitination of nearby monoubiquitinated Lys119 of histone H2A (H2AK119ub) to promote an open chromatin state. TET2 oxidizes m^5^C and antagonizes this MBD6-dependent H2AK119ub deubiquitination. TET2 depletion thereby leads to globally decreased H2AK119ub, more open chromatin and increased transcription in stem cells. *TET2-*mutant human leukaemia becomes dependent on this gene activation pathway, with *MBD6* depletion selectively blocking proliferation of *TET2*-mutant leukaemic cells and largely reversing the haematopoiesis defects caused by *Tet2* loss in mouse models. Together, our findings reveal a chromatin regulation pathway by TET2 through retrotransposon RNA m^5^C oxidation and identify the downstream MBD6 protein as a feasible target for developing therapies specific against *TET2* mutant malignancies.

## Abstract (web summary)

Chromatin-associated retrotransposon RNA 5-methylcytosine can be recognized by the methyl-CpG-binding-domain protein MBD6, which guides deubiquitination of nearby monoubiquitinated Lys119 of histone H2A to promote an open chromatin state in TET2-deficient cells.

## Main

**Extended Data Fig. 1:**
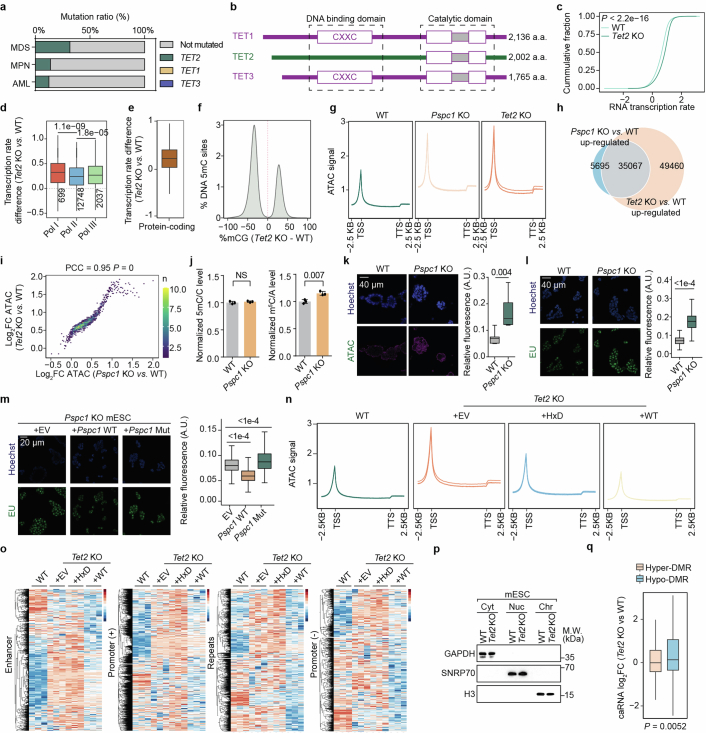
TET2 depletion led to global transcriptional activation in mESCs. **a**, The mutation profiles of TET family proteins (*TET1*, *TET2*, and *TET3*) in different diseases (from cbioportal.org). MDS, Myelodysplastic Syndromes; MPN, Myeloproliferative Neoplasms; AML, Acute Myeloid Leukaemia. **b**, Annotated domain structures of canonical human TET1-3 proteins. **c**, Nascent RNA transcription rate in WT and *Tet2-*KO mESCs. **d**, Transcription rates of genes regulated by RNA Polymerase I (Pol I), Polymerase II (Pol II), or Polymerase III (Pol III) in *Tet2*-KO *versus* WT mESCs defined using ChIP-seq data (GSE181698). **e**, Transcription rate changes of protein-coding genes in *Tet2*-KO *versus* WT mESCs. **f**, Distribution of 5mC methylation changes in *Tet2*-KO compared to WT mESCs at differentially methylated regions (DMRs) defined by Hon et al. (GSE48519). **g**, Metagene profile of calibrated ATAC-seq signals. **h**, Overlaps between ATAC-seq peaks with increased intensity for *Tet2*-KO *versus* WT, and for *Pspc1*-KO *versus* WT mESCs. **i**, Correlation of calibrated ATAC-seq signals comparing *Tet2*-KO with WT mESCs (y axis), and comparing *Pspc1*-KO with WT mESCs (x axis). PCC, Pearson correlation coefficient. **j**, DNA 5mC levels and caRNA m^5^C levels of WT and *Pspc1* KO mESCs. **k**-**m**, Representative images and quantifications measuring chromatin accessibility (**k**) and transcription rate (**l**) in WT and *Pspc1*-KO mESCs, or transcription rate (**m**) in *Pspc1*-KO mESCs overexpressing empty vector (EV), WT *Pspc1* (+*Pspc1* WT), or mutant *Pspc1* (+*Pspc1* Mut). Scale bar: 40 µm. ATAC: ATAC-see signal; EU: 5-ethynyl uridine; Hoechst: nucleus. **n**,**o**, Calibrated ATAC-seq signal in WT, as well as in *Tet2*-KO mESCs overexpressing either EV, wild-type mouse *Tet2* (WT), or catalytic dead mouse *Tet2* (HxD). Metagene profile (**n**) or heatmap showing signals on different carRNA regions (**o**). **p**, Representative western blot evaluation of chromatin preparations in mESCs. **q**, Changes in caRNA abundances at DMRs in *Tet2* KO *versus* WT mESCs. Box-plot elements: centre line, median; box limits, upper and lower quartiles; whiskers, 1–99% (**k**) or 1.5 × IQR (**d**,**e**,**l**,**m**,**q**). Bar represents mean and whiskers s.d. *P* values, two-tailed Wilcoxon-Mann-Whitney test (**c**,**d**,**l**,**m**,**q**), two-tailed unpaired *t* test with Welch’s correction (**j**,**k**), two-tailed t-distribution with n-2 degrees of freedom (**i**). NS, *P* > 0.05. *n* = 3 biological replicates (**g**,**k**-**q**). The depicted genome-wide data represent an integration of three biological replicates. Source Data

TET methylcytosine dioxygenases (TET1, TET2 and TET3) mediate oxidation of DNA 5-methylcytosine (5mC) to regulate gene expression in a wide range of different biological systems^6–8^. Among them, *TET2* is unique in that it distinctly exhibits high mutation ratios in myeloid malignancies (Extended Data Fig. 1a), with frequent *IDH* mutations observed in human cancers also thought to mainly act through TET2 inhibition^9–11^. TET2 deficiency led to genomic DNA hypomethylation^9^, suggesting that functional outcomes caused by TET2 deficiency might not primarily associate with its DNA oxidation activity. TET2 is also unique among TET enzymes in that it is not covalently linked to the zinc-finger CXXC domain protein CXXC4 or CXXC5^12–14^; the interaction between TET2 and CXXC4/CXXC5 is critical for DNA binding by TET2^15^ (Extended Data Fig. 1b). In mouse embryonic stem (mES) cells, it was shown that TET2 binds to PSPC1, an RNA-binding protein, to mediate RNA 5-methylcytosine (m^5^C) oxidation^16^. Other studies also reported RNA m^5^C oxidation by TET2 or *Drosophila* TET homologue^17–20^. We and others have recently reported chromatin regulation through reversible *N*^6^-methyladenosine modification on chromatin-associated RNA (caRNA)^21–25^. These advances prompted us to examine potential chromatin regulation through TET2-mediated caRNA m^5^C oxidation.

**Fig. 1:**
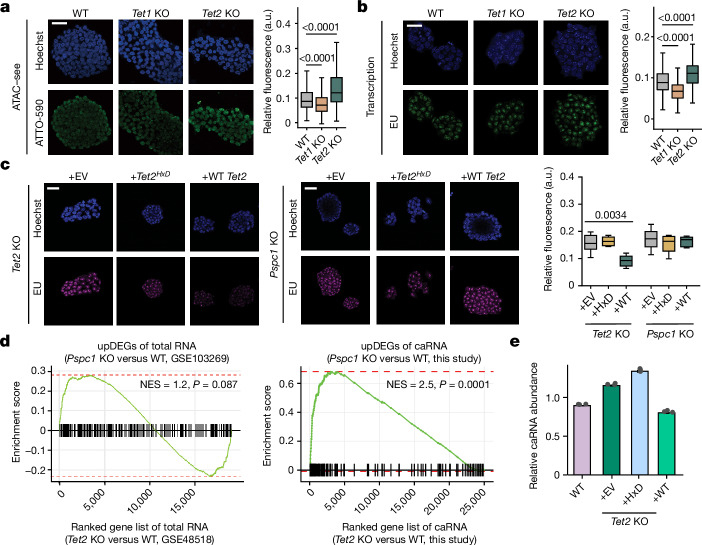
The elevated chromatin accessibility after *Tet2* depletion is facilitated by PSPC1 through its RNA-binding activity in mES cells. **a**,**b**, Representative immunofluorescence images and quantification characterizing chromatin accessibility (**a**) and nascent RNA synthesis rate (**b**) of WT and *Tet2*-KO mES cells. a.u., arbitrary units; ATAC–see, assay of transposase-accessible chromatin with visualization; EU, 5-ethynyl uridine. **c**, Representative immunofluorescence images characterizing the nascent RNA synthesis rate in *Tet2-*KO (left) or *Pspc1*-KO (right) mES cells, overexpressing either an EV control, WT mouse *Tet2* (WT) or catalytically dead mouse *Tet2* (HxD). For **a**–**c**, six images were taken for each condition. For **a**–**c**, scale bars, 40 μm. **d**, GSEA enrichment analysis between genes upregulated (upDEGs) after *Pspc1* depletion and upregulated DEGs after *Tet2* KO in mES cells. Left, whole-cell RNA-seq (Gene Expression Omnibus (GEO): GSE103269 and GSE48518). Right, caRNA-seq (this study). **e**, Spike-in-calibrated overall caRNA levels in WT mES cells, and *Tet2*-KO mES cells overexpressing either EV control, WT or HxD. Data are mean. For **a**–**c**, the box plots show the median (centre line), upper and lower quartiles (box limits) and 1–99% (whiskers). *P* values were calculated using two-tailed Wilcoxon–Mann–Whitney tests (**a** and **b**), two-tailed unpaired *t*-tests with Welch’s correction (**c**) and two-tailed permutation tests (**d**). *n* = 3 biological replicates (**a**–**c** and **e**). The depicted genome-wide data represent an integration of three biological replicates. Source Data

*Tet2*-knockout (KO) mES cells exhibited more open chromatin (Fig. 1a) and elevated global transcription (Fig. 1b) compared with the wild type (WT). Transcription rates of protein-coding genes were also higher in *Tet2*-KO mES cells when compared to those in WT (Extended Data Fig. 1c–e). The more-open chromatin state agrees well with the previously reported global DNA hypomethylation caused by TET2 deficiency^26^ (Extended Data Fig. 1f) but is inconsistent with a predominant DNA 5mC oxidation function.

To examine the functional outcomes of RNA oxidation by TET2, we studied *Pspc1-*KO mES cells. Spike-in-calibrated assay for transposase-accessible chromatin using sequencing (ATAC–seq) revealed globally increased chromatin accessibility in both *Pspc1*-KO and *Tet2-*KO mES cells (Extended Data Fig. 1g), with the more-opened chromatin loci notably overlapping and correlating with each other (Extended Data Fig. 1h,i). The DNA 5mC levels did not significantly change, while an increase in caRNA m^5^C levels was detected after *Pspc1* KO using ultra-high-performance liquid chromatography–tandem mass spectrometry (UHPLC–MS/MS) measurements (Extended Data Fig. 1j). The same chromatin openness and global transcription increases were observed in *Pspc1*-KO mES cells (Extended Data Fig. 1k,l). Overexpression of an RNA-binding-null PSPC1 mutant did not rescue these chromatin changes (Extended Data Fig. 1m). Moreover, overexpression of WT *Tet2*, but not its catalytic dead mutant (*Tet2*^*HxD*^, hereafter HxD)^27^, decreased the transcription rate and chromatin accessibility in *Tet2* KO, but not in *Pspc1*-KO mES cells (Fig. 1c). Global chromatin accessibility profiling also demonstrated a similar trend (Extended Data Fig. 1n,o). These results indicate that the TET2-mediated chromatin compaction and transcription repression are dependent on its enzymatic activity on RNA.

Consistent with the role of TET2 in suppressing transcription, the upregulation of caRNAs in *Pspc1*-KO mES cells, rather than whole-cell mRNAs, exhibited a stronger correlation with the changes observed after *Tet2* KO (Fig. 1d and Extended Data Fig. 1p). The elevated caRNA expression correlates with DNA hypomethylated regions instead of hypermethylated regions (Extended Data Fig. 1q). Overexpression of WT *Tet2*, but not HxD, was able to restore normal caRNA expression levels in *Tet2-*KO mES cells (Fig. 1e). Thus, while TET2 can act on either DNA or RNA, in mES cells, the TET2-mediated gene repression changes appear to be associated with its RNA-targeting activity.

## caRNA m^5^C oxidation by TET2

**Fig. 2:**
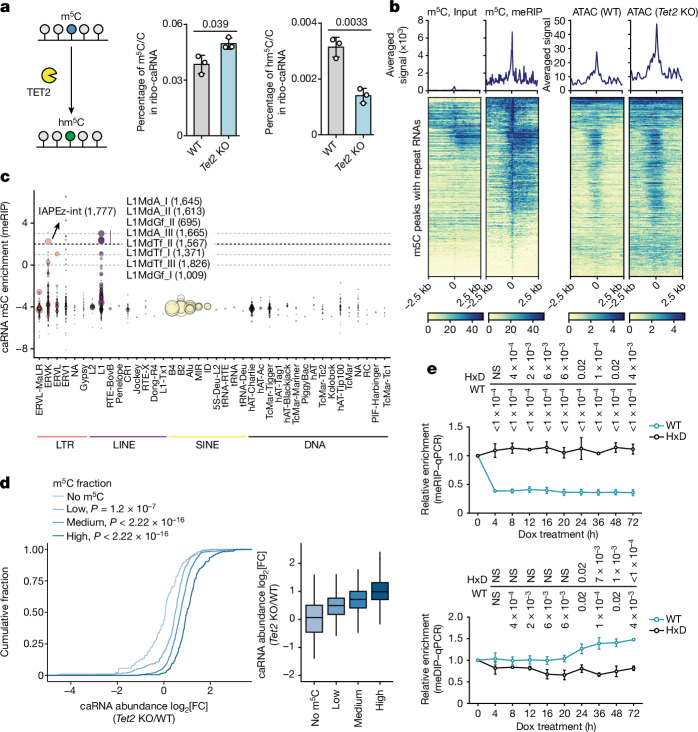
*Tet2* depletion led to elevated caRNA m^5^C methylation and abundance, resulting in local chromatin opening. **a**, The RNA m^5^C levels and 5-hydroxylmethylcytosine levels in the chromatin-associated fraction. m^5^C/C and hm^5^C/C values were obtained by normalizing absolute concentrations of m^5^C and hm^5^C to C. **b**, The average profile and heat map of the m^5^C level in WT mES cells, along with the corresponding ATAC–seq signals in WT and *Tet2-*KO mES cells on repeat RNA. The left colour bar shows meRIP signal and the right colour bar shows ATAC–seq signal. **c**, The m^5^C enrichment at various repeat RNA families. The size of each dot corresponds to the number of loci (subfamilies in ERVK or L1 shown) that were m^5^C methylated in WT mES cells. Exact loci numbers are indicated in parentheses. SINE, short interspersed nuclear element. **d**, Cumulative curve showing the log_2_-transformed fold change in repeat RNA expression after *Tet2* KO. Repeat RNAs were grouped on the basis of their m^5^C enrichment quantified by log_2_[IP/input] using m^5^C meRIP-seq data. No m^5^C, log_2_[IP/input] < 1; low, 1 ≤ log_2_[IP/input] < 2; medium: 2 ≤ log_2_[IP/input] < 3; high, log_2_[IP/input] ≥ 3. **e**, Local RNA m^5^C and DNA 5mC changes at IAP loci at different timepoints after DOX-induced dCas13–TET2-CD tethering. *P* values were determined by comparing values at the corresponding timepoints with values at 0 h, individually. qPCR, quantitative PCR. For **d**, the box plots show the median (centre line), the upper and lower quartiles (box limits) and 1.5× the interquartile range (whiskers). For **a** and **e**, data are mean ± s.d. *P* values were calculated using two-tailed unpaired *t-*tests with Welch’s correction (**a** and **e**). NS, *P* > 0.05. *n* = 3 biological replicates (**a** and **e**). The depicted genome-wide data represent an integration of three biological replicates. Source Data

**Extended Data Fig. 2:**
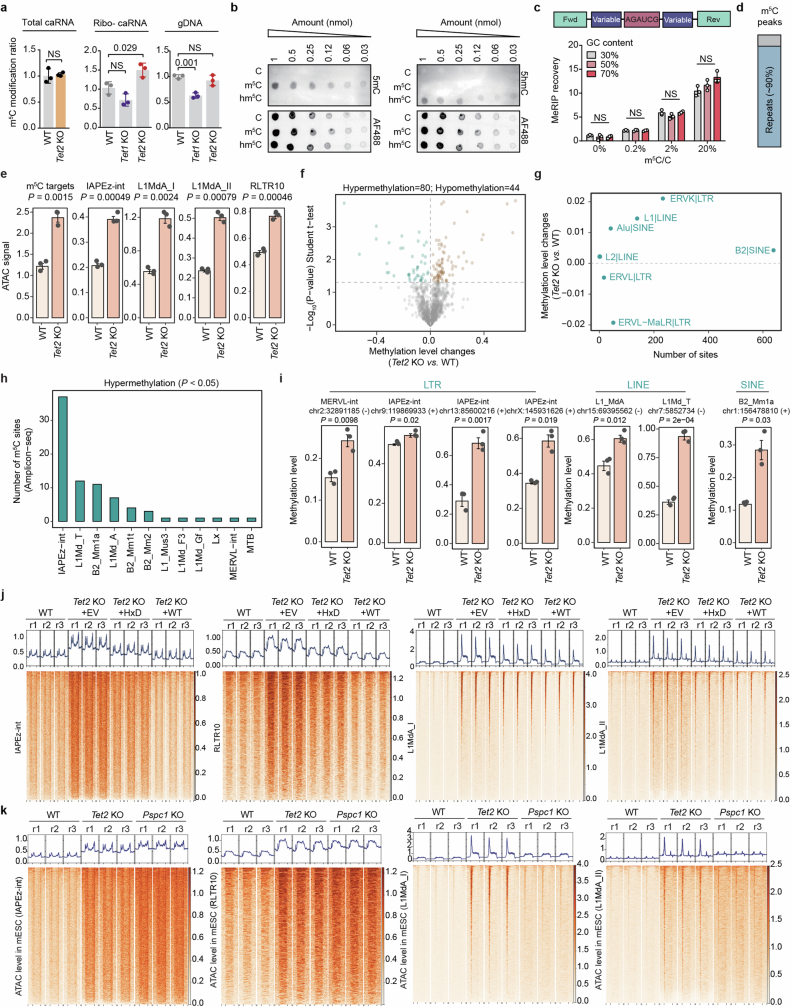
Repeat RNAs are major targets of TET2-mediated caRNA m^5^C oxidation. **a**, DNA 5mC and caRNA m^5^C levels. **b**, Representative imaging validating the binding specificity of the 5mC/5hmC antibody. AF488: total probe loading. Unmodified probe (C), m^5^C-modified probe (m^5^C), hm^5^C modified probe (hm^5^C). **c**, meRIP efficiency of RNA probes with different GC ratio and m^5^C modification levels. Comparisons are within the same m^5^C/C ratio group. **d**, Ratio of caRNA m^5^C meRIP peaks on repeat or non-repeat RNA. **e**, Calibrated global ATAC-seq signals across different repeat RNA families and m^5^C sites. **f**, Changes in RNA m^5^C methylation levels upon *Tet2* KO and their -log_10_(p-value). **g**, Number of methylated sites and the changes in methylation levels for each repeat RNA family following *Tet2* KO in mESCs. Each dot represents one repeat RNA family, with the number of methylated sites counted and the changes in methylation levels averaged within each family. **h**, Number of hypermethylated sites located across different repeat RNA families. **i**, m^5^C methylation levels on different repeat RNA loci in WT and *Tet2*-KO mESCs. **j**, Heatmap showing calibrated ATAC-seq signals in WT, as well as in *Tet2*-KO mESCs overexpressing either an empty vector (EV) control, wild-type mouse *Tet2* (WT), or catalytically inactive mouse *Tet2* (HxD), across different repeat RNA families including IAPEz-int, RLTR10, L1MdA_I, and L1MdA_II. **k**, Heatmap showing calibrated ATAC-seq signals in WT, *Pspc1*-KO and *Tet2*-KO mESCs, across different repeat RNA families including IAPEz-int, RLTR10, L1MdA_I, and L1MdA_II. Bar represents mean and whiskers s.d. (**a**,**c**) or s.e.m (**e**,**i**). *P* values, One-Way ANOVA (**c**), two-tailed unpaired *t* test with Welch’s correction (**a**,**e**,**f**,**h**,**i**). NS, *P* > 0.05. *n* = 3 biological replicates (**a**-**c**, **e**-**k**). The depicted genome-wide data represent an integration of three biological replicates. Source Data

We next investigated whether m^5^C on caRNA is a substrate of TET2. UHPLC–MS/MS identified m^5^C in ribosomal RNA (rRNA)-depleted caRNAs (Fig. 2a). *Tet2* KO led to a notable increase in caRNA m^5^C level, accompanied by a decrease in the levels of its oxidation product 5-hydroxymethylcytosine (hm^5^C) (Fig. 2a and Extended Data Fig. 2a).

We profiled caRNA m^5^C by methylated RNA immunoprecipitation followed by sequencing (meRIP–seq; Extended Data Fig. 2b,c). Most of the caRNA m^5^C peaks are in repeat RNA (Extended Data Fig. 2d). These m^5^C-marked repeat RNAs are associated with increased local chromatin accessibility (Fig. 2b and Extended Data Fig. 2e), with the long terminal repeat (LTR) and long interspersed nuclear element (LINE) families mostly enriched (Fig. 2c). We also performed quantitative amplicon sequencing analysis of selected amplicons among these repeat RNAs after ultrafast bisulfite treatment^28^. We observed more caRNA m^5^C hypermethylation in these amplicons from *Tet2*-KO mES cells (Extended Data Fig. 2f–i). ERVK and L1 families bear the most hypermethylation sites (Extended Data Fig. 2h). We examined ERVK and L1 with IAPEz-int/RLTR10 and L1MdA_I/II subfamilies as their respective representatives because they are the top-ranked subfamilies observed in the m^5^C meRIP enrichment. Compared with empty vector (EV) control or HxD, overexpressing WT *Tet2* caused notable decreases in chromatin accessibility in these repeat regions (Extended Data Fig. 2j). While intracisternal A particle (IAP) displayed an increase in local chromatin accessibility after either *Tet2* or *Pspc1* KO (Extended Data Fig. 2k), LINE1-associated chromatin accessibility increased only after *Tet2* KO but not *Pspc1* deletion (Extended Data Fig. 2k), suggesting that LINE1-associated chromatin changes may depend on TET2 in a PSPC1-independent manner.

**Extended Data Fig. 3:**
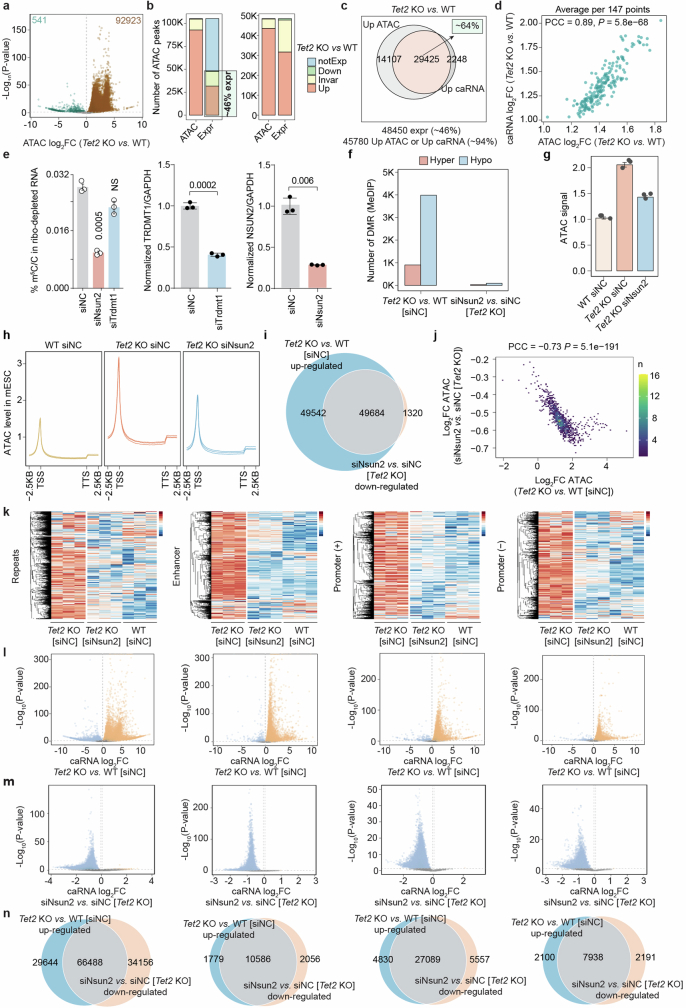
NSUN2 installs caRNA m^5^C and regulates chromatin openness. **a**, Changes in ATAC-seq peak intensity of *Tet2*-KO *versus* WT mESCs. **b**, Left panel: Total number of ATAC-seq peaks in mESCs. Right panel: Number of ATAC-seq peaks associated with caRNA expression in mESCs. These peaks were categorized into four groups: differentially upregulated (Up), downregulated (Down), or unchanged (Invar) upon *Tet2* KO in mESCs. Additionally, they were classified based on their transcriptional status as either transcribed (Expr) or not transcribed (notExp). **c**, Overlap of ATAC-seq peaks with increased intensity (up ATAC) or increased caRNA abundance in *Tet2*-KO mESCs. **d**, Correlation of changes in chromatin accessibility and caRNA abundance upon *Tet2* KO in mESCs. **e**, m^5^C levels in caRNAs from WT mESCs transfected with non-targeting siRNA (siNC), or siRNAs targeting *Nsun2* or *Trdmt1*. KD efficacies of *Trdmt1* or *Nsun2* in mESCs. Comparisons against siNC group. **f**, Number of DMRs identified in *Tet2*-KO compared to WT mESCs, and in *Nsun2* KD compared to control *Tet2*-KO mESCs. **g**, Calibrated ATAC-seq signals in WT, *Tet2*-KO, and *Nsun2-*KD *Tet2*-KO mESCs. **h**, Metagene Profile of calibrated ATAC-seq signals in WT, *Tet2*-KO, and *Nsun2*-KD *Tet2*-KO mESCs. **i**, Overlap of ATAC-seq peaks with increased intensity in *Tet2*-KO *versus* WT mESCs, and ATAC-seq peaks with decreased intensity in *Nsun2*-KD compared with control *Tet2*-KO mESCs. **j**, Correlation of changes in calibrated ATAC-seq signals comparing *Tet2*-KO with WT mESCs, and comparing *Nsun2*-KD with control *Tet2*-KO mESCs. **k**, caRNA expression profiles in WT, *Tet2*-KO mESCs, and in *Nsun2*-KD *Tet2*-KO mESCs, across different carRNA species. **l**-**m**, Volcano plots of calibrated caRNA expression changes comparing *Tet2*-KO to WT mESCs (**l**), and in *Nsun2* KD compared to control *Tet2*-KO mESCs (**m**). **n**, Overlap of differentially expressed caRNAs in *Tet2*-KO compared to WT, and in *Nsun2*-KD compared to control *Tet2*-KO mESCs. Bar represents mean and whiskers s.d. (**e**) or s.e.m (**g**). *P* values, Wald-test (**a**,**l**,**m**), two-tailed unpaired *t* test with Welch’s correction (**e**), two-tailed t-distribution with n-2 degrees of freedom (**d**,**j**). NS, *P* > 0.05. *n* = 3 biological replicates (**e**,**g**,**h**,**k**). The depicted genome-wide data represent an integration of three biological replicates. Source Data

We further analysed the correlation between caRNA expression changes with ATAC signals after *Tet2* KO. *Tet2* KO resulted in a global increase in chromatin accessibility (Extended Data Fig. 3a). While caRNA transcription only occurred at 46% of chromatin regions with identified ATAC peaks (Extended Data Fig. 3b), 60% of those with a more opened chromatin state also exhibited an increase in caRNA level after *Tet2* KO (Extended Data Fig. 3c). Changes in chromatin accessibility correlate well with caRNA increases in these regions (Extended Data Fig. 3d). m^5^C-marked caRNA abundance showed even greater increases after *Tet2* KO (Fig. 2d). These collectively suggest that chromatin-associated regulatory RNA (carRNA) m^5^C methylation regulates local chromatin accessibility.

NOP2/Sun RNA methyltransferase 2 (NSUN2) and DNA methyltransferase 2 (TRDMT1) are likely candidates that may install caRNA m^5^C, as both are known to localize in the cell nucleus and mediate RNA m^5^C methylation^29,30^. Only *Nsun2* depletion caused an approximately 70% decrease in caRNA m^5^C abundance (Extended Data Fig. 3e) without affecting the DNA 5mC levels (Extended Data Fig. 3f). *Nsun2* knockdown (KD) also led to a more-closed chromatin state (Extended Data Fig. 3g,h). These changed regions largely overlap and negatively correlate with the more opened chromatin loci in *Tet2-*KO mES cells (Extended Data Fig. 3i,j). Moreover, transcriptome-wide alterations caused by *Nsun2* depletion exhibited patterns that contrast the gene expression changes caused by *Tet2* depletion (Extended Data Fig. 3k–n).

**Extended Data Fig. 4:**
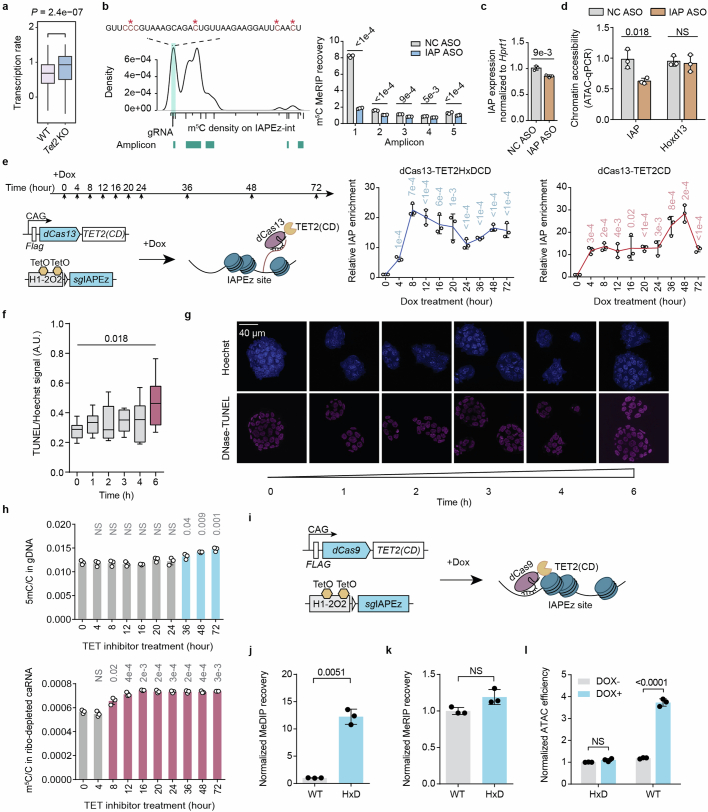
RNA oxidation by TET2 leads to chromatin compaction. **a**, IAPEz-nearby gene transcription in WT and *Tet2*-KO mESCs. **b**, Metagene profiles of IAP RNA m^5^C landscape in WT mESCs with individual amplicons indicated, and zoom-in showing the 5′ end sequence that contains multiple m^5^C sites revealed by bisulfite sequencing. meRIP-qPCR with primers targeting different amplicons enabled region-specific analysis of the IAP m^5^C level upon IAP ASO treatment in mESCs. IAP ASO targets amplicon 1, a non-targeting ASO (NC ASO) was used as control. **c**, IAP ASO treatment slightly downregulated IAP RNA level in mESCs. **d**, Chromatin accessibility at IAP loci following ASO treatment. **e**, Schematics showing the design of CRISPR tethering system of the TET2 catalytic domain to IAP RNA. mESCs stably express dCas13-TET2(CD) or a catalytic dead version (dCas13-TET2(CD)-HxDmut) fusion protein and guide RNA sequence downstream of a TetO-controlled H1 promotor (H1-2O2). Dox-inducible tethering to IAP is validated by RIP-qPCR. Comparisons against “0 h” group. **f**-**g**, Quantification (**f**) and representative immunofluorescence images (**g**) for DNase-TUNEL assay in mESCs treated by TET inhibitor TETi-C35. Scale bar: 40 μm. Comparison between “6 h” and “0 h” groups. **h**, Abundance of DNA 5mC in genomic DNA or RNA m^5^C in caRNA measured in mESCs at indicated time points post TETi-C35 treatment. Comparisons against “0 h” group. **i**, Schematics showing the design of CRISPR tethering system of the TET2 catalytic domain (TET2(CD)) to IAP genomic loci with a similar design as (**e**). **j**-**l**, DNA 5mC changes (**j**), RNA m^5^C changes (**k**) or chromatin accessibility changes (**l**) at IAP loci when dCas9-TET2CD fusion protein was targeted to IAP loci with guide RNA. HxD represents the catalytic dead mutant, WT represents wild-type TET2 catalytic domain. Box-plot elements: centre line, median; box limits, upper and lower quartiles; whiskers, 1–99% (**a**,**f**). Bar (**b**-**d**,**h**,**j**-**l**) or line centre (**e**) represents mean and whiskers s.d. *P* values, two-tailed Wilcoxon-Mann-Whitney test (**a**,**f**), two-tailed unpaired *t* test with Welch’s correction (**b**-**e**,**h**,**j**-**l**). NS, *P* > 0.05. *n* = 3 biological replicates (**b**-**l**). The depicted genome-wide data represent an integration of three biological replicates. Source Data

Purified TET2 protein is known to mediate oxidation of RNA m^5^C to hm^5^C^18^. We also identified an increase in caRNA m^5^C in *Tet2-*KO mES cells (Fig. 2a), with IAP RNA from the LTR family as a notable example (Fig. 2c). IAP also had most hypermethylated m^5^C sites in response to *Tet2* KO (Extended Data Fig. 2g–i). *Tet2* depletion led to increased caRNA IAP m^5^C levels (Extended Data Fig. 2f–i), increased local chromatin accessibility (Extended Data Fig. 2j,k) and accelerated transcription of its target RNAs (Extended Data Fig. 4a), suggesting that IAP RNA is a main downstream regulator of the TET2-mediated chromatin regulation in mES cells.

We next blocked IAP RNA methylation using an anti-sense oligo (ASO) targeting its main m^5^C site (Extended Data Fig. 4b). This ASO selectively blocked IAP RNA m^5^C installation (Extended Data Fig. 4b), with IAP RNA levels remaining almost unchanged (Extended Data Fig. 4c). Administration of this ASO led to more closed local chromatin at IAP loci (Extended Data Fig. 4d). Thus, m^5^C methylation on chromatin-associated IAP RNA could regulate the local chromatin state.

To establish the key causal relationship, we constructed the locus-specific RNA-targeting system (Extended Data Fig. 4b) by fusing dCas13 with the TET2 catalytic domain (TET2-CD). The dCas13–TET2-CD fusion protein was stably expressed with the guide RNA under the control of a doxycycline (DOX)-responsive Tet operator (Extended Data Fig. 4e). Acute expression of the guide RNA caused rapid dCas13–TET2-CD recruitment and reduction of RNA m^5^C methylation on IAP transcripts, followed by increased local DNA 5mC methylation (Fig. 2e and Extended Data Fig. 4e). By contrast, tethering of HxD did not alter DNA or RNA methylation, demonstrating that this effect is dependent on its oxidation activities but not the protein scaffolding effect^31^. The increased DNA 5mC methylation caused by TET2 targeting to RNA agrees with the widespread DNA hypomethylation or chromatin opening after TET2 inactivation frequently observed in embryonic stem cells, haematopoietic stem cells and cancer cells^5^. We therefore conclude that the global chromatin and transcriptional regulation effects of TET2 are most likely mediated through RNA m^5^C oxidation.

To further confirm this enzymatic-activity-dependent regulation, we treated mES cells with an inhibitor against TET enzymes^32^. Time-lapse tracking after treatment revealed early chromatin opening (Extended Data Fig. 4f,g) and caRNA m^5^C increases (Extended Data Fig. 4h (bottom)), followed by genomic DNA 5mC increases (Extended Data Fig. 4h (top)). These changes in chromatin state were most likely due to the RNA oxidation activity of TET2. By contrast, tethering TET2-CD to IAP loci using DNA-binding dCas9 led to expected DNA hypomethylation and increased chromatin accessibility (Extended Data Fig. 4i–l).

**Fig. 3:**
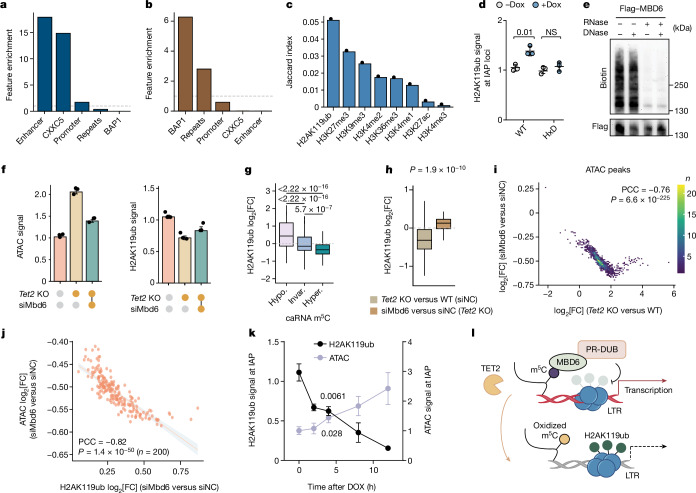
The activity of TET2 on RNA dictates the increased chromatin accessibility through MBD6-mediated H2AK119ub deubiquitination. **a**,**b**, Feature enrichment in hypermethylated differentially methylated regions (DMRs) compared with hypomethylated DMRs (hyperspecific; **a**), and in hypomethylated DMRs compared with hypermethylated DMRs (hypospecific; **b**) using the odds ratio. **c**, Overlapping ratios of histone modifications with DNA hypomethylated regions for *Tet2*-KO versus WT mES cells. **d**, The H2AK119ub levels at IAP loci after TET2-CD (catalytic domain; WT or catalytically dead HxD) tethering by dCas13. **e**, Representative image showing nucleic acids cross-linked to MBD6. **f**, Spike-in-calibrated ATAC–seq signals or H2AK119ub signals in WT (siNC WT) and *Tet2-*KO (siNC *Tet2* KO) mES cells, as well as in *Mbd6-*KD *Tet2*-KO (siMbd6 *Tet2* KO) mES cells. **g**, Changes in H2AK119ub on m^5^C hypomethylated (hypo.), unchanged (invar.) and hypermethylated (hyper.) repeat RNA after *Tet2* KO versus WT. **h**, H2AK119ub changes in m^5^C hypermethylated repeat RNA in *Tet2*-KO versus WT compared with *Mbd6-*KD *Tet2*-KO versus *Tet2*-KO mES cells. **i**, The correlation between ATAC signal fold changes in *Tet2*-KO versus WT mES cells, and comparing *Mbd6* KD with control *Tet2*-KO mES cells. PCC, Pearson correlation coefficient. **j**, The correlation between changes in ATAC and H2AK119ub signals, comparing *Mbd6* KD with control *Tet2-*KO mES cells. **k**, The H2AK119ub levels and ATAC signal at IAP RNA after dCas13–MBD6-MBD tethering. **l**, Schematics of the proposed pathway of MBD6 regulating chromatin state through caRNA m^5^C binding. Data are mean ± s.d. (**d** and **k**) and mean ± s.e.m. (**f**). For **g** and **h**, the box plots show the median (centre line), the upper and lower quartiles (box limits) and 1.5× the interquartile range (whiskers). *P* values were calculated using two-tailed unpaired *t*-tests with Welch’s correction (**d** and **k**), two-tailed Wilcoxon–Mann–Whitney tests (**g** and **h**), two-tailed *t*-distribution with *n* − 2 d.f. (**i** and **j**). *n* = 3 biological replicates (**d**–**f** and **k**). The depicted genome-wide data represent an integration of three biological replicates. Source Data

**Extended Data Fig. 5:**
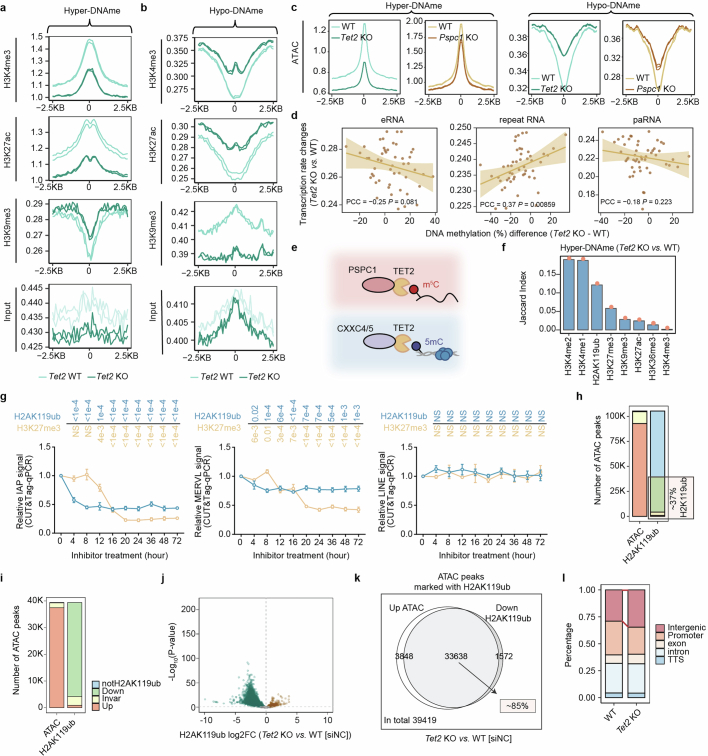
H2AK119ub modification is critical to TET2-mediated chromatin regulation. **a**,**b**, Average histone modification levels, as well as input signals, in WT and *Tet2*-KO mESCs at DNA hypermethylated regions (**a**) or hypomethylated regions (**b**), detected in *Tet2*-KO mESCs. **c**, Average ATAC signals in WT, *Tet2*-KO, and *Pspc1*-KO mESCs at DNA 5mC hypermethylated regions or hypomethylated regions detected in *Tet2*-KO mESCs. **d**, Correlation between DNA methylation differences in *Tet2*-KO *versus* WT mESCs and changes in their downstream gene transcription. carRNAs were categorized into different groups, including eRNA, paRNA, and repeat RNA. Within each group of carRNAs, they were further divided into 50 bins based on the ranked DNA methylation differences upon *Tet2* KO in mESCs. Error band represents standard errors. **e**, Schematics showing the dual mode of chromatin regulation by TET2 when bound to different protein partners. **f**, Overlapping ratios of regions marked by different histone modifications with DNA hypermethylated regions (detected in the *Tet2*-KO mESCs). **g**, H2AK119ub and H3K27me3 chromatin bindings at IAP, MERVL, and LINE loci measured through CUT&Tag protocol. Comparisons against “0 h” group. **h**-**i**, Total number of ATAC-seq peaks in mESCs. **h**, Number of ATAC-seq peaks marked with H2AK119ub modification in mESCs; **i**, ATAC-seq peaks were categorized into four groups: differentially upregulated (Up), downregulated (Down), or unchanged (Invar) after *Tet2* KO in mESCs. Additionally, they were classified based on their association with H2AK119ub as either marked (H2AK119ub) or not marked (notH2AK119ub). **j**, Volcano plot displaying changes in H2AK119ub peak intensity for *Tet2*-KO versus WT mESCs. **k**, Overlap of ATAC-seq peaks with increased chromatin accessibility (up ATAC) or decreased H2AK119ub (Down H2AK119ub) in *Tet2*-KO mESCs. **l**, Genomic distribution of H2AK119ub peaks in WT and *Tet2*-KO mESCs. Line centre (**g**) represents mean and whiskers s.d. *P* values, Wald test (**j**), two-tailed unpaired *t* test with Welch’s correction (**g**), two-tailed t-distribution with n-2 degrees of freedom (**d**). NS, *P* > 0.05. *n* = 3 biological replicates (**g**). The depicted genome-wide data represent an integration of three biological replicates (**c**,**d**,**f**,**h**-**l**) or two biological replicates (**a**,**b**). Source Data

DNA hypermethylated and hypomethylated regions were both found in *Tet2*-KO mES cells (Extended Data Fig. 1f), accompanied by changes in chromatin accessibility in the opposite directions (Extended Data Fig. 5a–c). The increased caRNA expression was observed only in DNA hypomethylated regions after *Tet2* KO in mES cells (Extended Data Fig. 1q). To separate effects on DNA versus RNA, we further analysed DNA 5mC changes caused by *Tet2* KO at different genomic regions, including enhancers, promoters and repeats (Extended Data Fig. 5d). We observed a negative correlation between changes in enhancer transcription and DNA methylation resulting from *Tet2* KO in mES cells, which contrasts with the pattern observed for repeat RNA (Extended Data Fig. 5d). Furthermore, these DNA hypermethylated regions resulting from *Tet2* KO were enriched at enhancer and CXXC5-bound regions while being depleted in repeats (Fig. 3a), agreeing with the notion that DNA 5mC oxidation by TET2 in these enhancer regions leads to local transcription activation.

In contrast to enhancer regions, repeat loci were enriched in DNA hypomethylated regions after *Tet2* depletion (Fig. 3b). Moreover, these DNA hypomethylated regions were more accessible after *Tet2* or *Pspc1* KO (Extended Data Fig. 5c). These suggest that the elevated chromatin accessibility resulting from TET2 depletion cannot be attributed to its 5mC oxidation activity in DNA but, rather, its oxidation activity on RNA m^5^C. We conclude that TET2 can mediate either DNA 5mC or RNA m^5^C oxidation by engaging different protein partners (Extended Data Fig. 5e). It is the repeat RNA (for example, LTR RNA) m^5^C oxidation that causes chromatin compaction, which dominates chromatin and transcription regulation in mES cells.

## MBD6 binds to RNA m^5^C to recruit PR-DUB

We computationally analysed histone modifications that correlate best with the m^5^C oxidation by TET2 on RNA. We found that H2AK119ub, a major chromatin repressive mark installed by PRC1^33^, ranked as the top (Fig. 3c and Extended Data Fig. 5f). H2AK119ub can be erased by the polycomb repressive deubiquitylase (PR-DUB) complexes^33–36^, and we also identified BAP1 (core component of PR-DUB) binding as one top enriched genomic feature (Fig. 3b). Consistently, tethering of WT dCas13–TET2-CD, but not HxD, increased H2AK119ub at the IAP loci (Fig. 3d). Time-lapse tracking of H2AK119ub and H3K27me3 marks^37^ at IAP and control LINE loci after acute TET inhibition also showed an early response in H2AK119ub (Extended Data Fig. 5g). We next profiled H2AK119ub changes with and without *Tet2* deletion. Approximately 37% of ATAC–seq peaks were marked with H2AK119ub (Extended Data Fig. 5h), among which H2AK119ub was downregulated in around 89% of these genomic regions (Extended Data Fig. 5i,j). Moreover, approximately 85% of genomic regions with higher ATAC signals overlapped well with H2AK119ub loss (Extended Data Fig. 5k,l). Thus, most of the opened chromatin regions caused by *Tet2* KO display decreased H2AK119ub, which may initiate changes in local chromatin accessibility and transcription.

**Extended Data Fig. 6:**
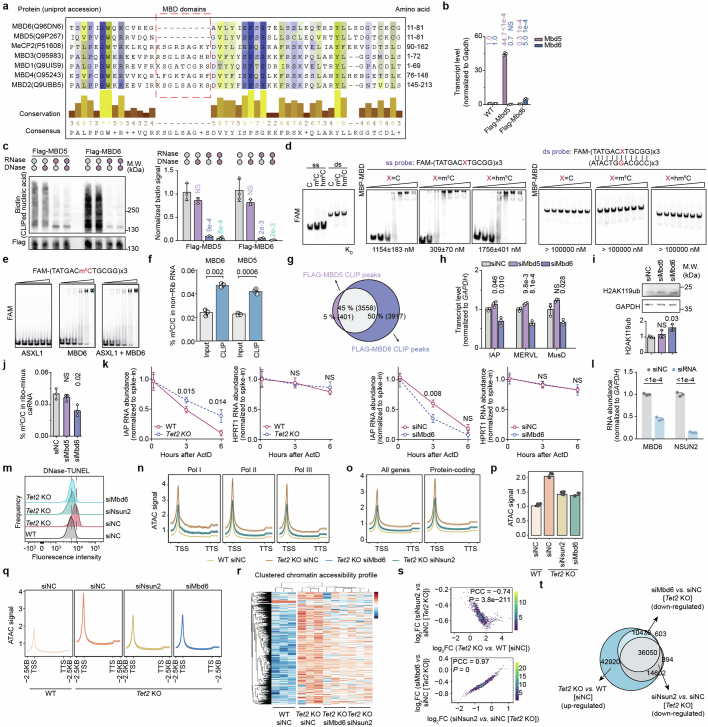
MBD6 preferentially binds to m^5^C-modified RNA and regulates chromatin H2AK119ub levels. **a**, Sequence alignment of human MBD family proteins (MBD1-6 and MeCP2) showing differences in the MBD region. **b**, Validation of Flag-MBD5 or Flag-MBD6 overexpressing mESCs using RT-qPCR. Averaged transcript expressions were indicated. Comparisons against “WT” group. **c**, Representative gel image quantifying nucleic acids crosslinked to MBD5 or MBD6. Comparisons against “RNase-, DNase-” group. **d**, EMSA showing the binding preferences of the purified MBD domain of MBD6 fused to maltose binding protein (MBP) with C, m^5^C, or hm^5^C-containing oligonucleotide probes. The purity of the single-stranded probe (ss probe) and double-stranded probe (ds probe) was validated by electrophoresis. Gradient for MBP-MBD protein was 0, 0.1, 0.5, 2.5, 12, 50, and 100 μM. **e**, EMSA showing that ASXL1 binding to MBD6 does not affect MBD6-m^5^C binding. MBD6 MBD domain (amino acid 1–100 fused by MBP-His) and ASXL1 ASXH domain (amino acid 1509–1541 fused by His-SUMO) were used. **f**, m^5^C abundance in MBD5 and MBD6 enriched RNA. **g**, Overlap between the RNA binding sites of MBD5 and MBD6. **h**-**j**, Repeat RNA expression (**h**), H2AK119ub (**i**) or caRNA m^5^C (**j**) level upon *Mbd5* or *Mbd6* KD. Comparisons against “siNC” group. **k**, IAP RNA lifetime upon *Tet2* KO or *Mbd6* KD in mESCs. **l**, qPCR results assessing *Mbd6* or *Nsun2* RNA abundance in mESCs. Comparisons against “siNC” groups. **m**, Chromatin openness measured by DNase-TUNEL assay upon *Nsun2* or *Mbd6* KD. **n**-**r**, Spike-in calibrated ATAC-seq signals in WT (WT [siNC]) and *Tet2* KO (*Tet2* KO [siNC]) mESCs, as well as *Mbd6* KD (*Tet2* KO siMbd6) and *Nsun2* KD (*Tet2* KO siNsun2) in *Tet2*-KO mESC cells. Metagene profile at genes regulated by different RNA polymerases (**n**), protein-coding genes (**o**), overall signal (**p**,**q**), or clustered ATAC peaks (**r**) were shown. **s**, Correlation fold changes in spike-in calibrated ATAC-seq signals in *Tet2* KO *versus* WT mESCs, and comparing *Nsun2* KD or *Mbd6* KD with control *Tet2* KO mESCs. **t**, Venn diagram displaying the significantly up-regulated ATAC-seq peaks in *Tet2* KO compared with WT mESC cells, and down-regulated ATAC-seq peaks in *Nsun2* KD or *Mbd6* KD compared with control mESC cells following *Tet2* KO. Bar (**b**,**c**,**f**,**h**-**j**,**l**,**p**) or line centre (**k**) represents mean and whiskers s.d. *P* values, two-tailed unpaired *t* test with Welch’s correction (**b**,**c**,**f**,**h**-**l**), two-tailed t-distribution with n-2 degrees of freedom (**s**). NS, *P* > 0.05. *n* = 3 biological replicates (**b**-**f**,**h**-**r**). The depicted genome-wide data represent an integration of three biological replicates. Source Data

Previous studies have identified MBD5 and MBD6 as partner proteins of PR-DUB, and their localization to heterochromatin appeared to be independent of DNA 5mC^38,39^. MBD5 and MBD6 both possess a conserved but structurally distinct methyl-binding domain (MBD) but do not bind to DNA^38^ (Extended Data Fig. 6a). We speculated that these two proteins might bind to RNA m^5^C, which may then recruit PR-DUB to mediate H2AK119ub deubiquitination at the m^5^C-methylated LTR loci for transcription activation.

We examined nucleic acids bound by MBD5 or MBD6 in mES cells (Extended Data Fig. 6b). We observed cross-linked RNA but not DNA, as an RNase treatment almost completely abolished nucleic acid signals, while the effect of a DNase treatment was minor (Fig. 3e and Extended Data Fig. 6c). The purified MBD domain of MBD6 preferentially bound to a single-stranded oligonucleotide probe containing m^5^C over unmethylated or hm^5^C-modified probe (Extended Data Fig. 6d), and this binding was not affected by the binding of MBD6 to ASXL1 (Extended Data Fig. 6e), a protein that bridges MBD6 with PR-DUB^40^. Cellular MBD5 and MBD6 also enrich m^5^C-containing RNA (Extended Data Fig. 6f). Thus, our data revealed that MBD5 and MBD6 are RNA-binding proteins that preferentially recognize RNA m^5^C.

While the RNA-binding targets of MBD5 and MBD6 substantially overlap with each other (Extended Data Fig. 6g), MBD6 appears to dominate the regulation of H2AK119ub and repeat RNA expression in mES cells, as KD of *Mbd6* was sufficient to reverse elevated expression of LTRs caused by *Tet2* KO, whereas *Mbd5* KD did not do so (Extended Data Fig. 6h). The global H2AK119ub levels also significantly increased only after *Mbd6* KD (Extended Data Fig. 6i). *Mbd6* KD caused a global decrease in the caRNA m^5^C levels (Extended Data Fig. 6j). Consistent with this, IAP RNAs were stabilized in *Tet2*-KO mES cells and were destabilized after *Mbd6* KD (Extended Data Fig. 6k). m^5^C methylation appears to stabilize LTR RNAs and this effect is mediated largely through MBD6. We therefore focused on MBD6 in subsequent studies, although MBD5 may have important roles in other cell types.

Functionally, *Mbd6* KD was able to rescue the genome-wide increased chromatin accessibility (Fig. 3f (left)) and decreased the H2AK119ub levels (Fig. 3f (right)) in *Tet2-*KO mES cells. Consistently, we observed decreased H2AK119ub at caRNA m^5^C hypermethylated sites after *Tet2* deletion (Fig. 3g), and this change could be largely reversed by *Mbd6* KD (Fig. 3h). *Mbd6* or *Nsun2* KD (Extended Data Fig. 6l) both decreased chromatin openness caused by *Tet2* KO in mES cells (Extended Data Fig. 6m), with consistent global decreases in chromatin accessibility (Extended Data Fig. 6n–r). Moreover, genomic regions with altered chromatin openness overlap and correlate well between different groups (Fig. 3i and Extended Data Fig. 6s,t; *Tet2* KO versus WT, small interfering RNA against *Mbd6* (siMbd6) versus siNC in *Tet2* KO, and siNsun2 versus siNC in *Tet2* KO). Local chromatin accessibility changes in response to *Mbd6* KD exhibit a negative correlation with the resulting H2AK119ub increases (Fig. 3j). Acute recruitment of dCas13 fused to the methyl-CpG binding domain of MBD6 protein (dCas13–MBD6-MBD) was also sufficient to reduce local H2AK119ub and induce open chromatin at IAP loci (Fig. 3k).

Together, our results reveal that TET2-mediated caRNA m^5^C oxidation reduces both MBD6 binding and local histone H2AK119ub deubiquitination, leading to closed chromatin and transcription suppression (Fig. 3l). Whether the oxidation product hm^5^C may further promote caRNA degradation remains to be investigated in the future.

## Targeting MBD6 in TET2-deficient HSPCs

**Fig. 4:**
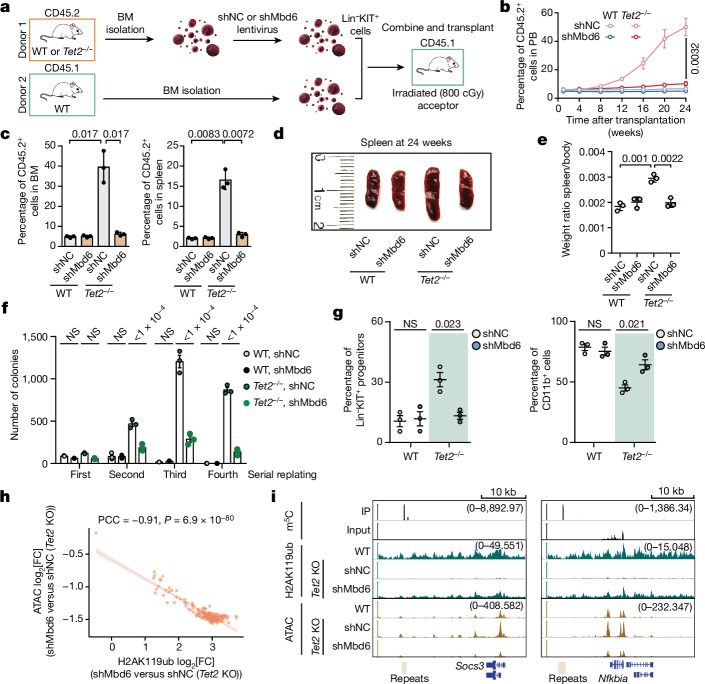
Targeting MBD6–m^5^C pathway rescues haematopoietic defects caused by *Tet2* deletion in HSPCs. **a**, Schematics of the mixed chimera transplantation assay. **b**, Quantification of CD45.2^+^ cells in the PB of recipient mice at different timepoints after transplantation. **c**, Quantification of CD45.2^+^ cells in the BM (left) or spleen (right) of recipient mice. **d**,**e**, Representative image (**d**) and quantification (**e**) of spleen size isolated from recipient mice at 24 weeks after transplantation. **f**,**g**, Colony formation analysis of the serial replating assay (**f**) and flow cytometry analyses of suspension cultures (**g**) of WT and *Tet2*^*−/−*^ HSPCs with (shMbd6) or without (shNC) *Mbd6* KD. **h**, The correlation between changes in ATAC–seq signal and H2AK119ub signal in response to *Mbd6* KD in *Tet2*-KO HSPCs. **i**, Integrative Genome Viewer visualization of the H2AK119ub and ATAC signal around the *Socs3* or *Nfkbia* genes in WT, *Tet2* KO and shMbd6 after *Tet2* KO in HSPCs. The values in parentheses represent the scale of signal in each track. Data are mean ± s.d. (**b**, **c** and **e**–**g**). *P* values were calculated using two-tailed unpaired *t*-tests with Welch’s correction (**b**, **c** and **e**–**g**) and two-tailed *t*-distribution with *n* − 2 d.f. (**h**). *n* = 3 biological replicates (**b**–**g**). The depicted genome-wide data represent an integration of three biological replicates. Source Data

**Extended Data Fig. 7:**
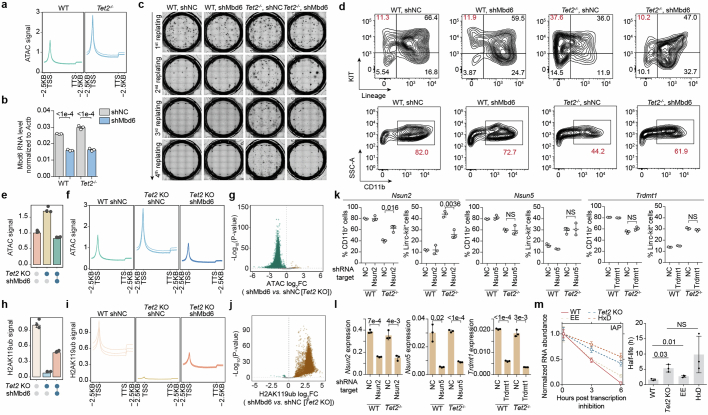
*Mbd6* KD restored the TET2-loss-mediated abnormal HSPC proliferation and differentiation through carRNA m^5^C. **a**, Metagene profile of the spike-in calibrated ATAC-seq signals of WT and *Tet2*^*−/−*^ HSPCs. **b**, qPCR results validating efficiency of shRNA-enabled *Mbd6* KD in HSPCs. **c**, Colony formation of serial replating assays of HSPCs upon *Mbd6* KD. **d**, Representative flow cytometry results characterizing HSPC frequency in suspension cultures (top, KIT and Lineage as markers, day 7) and differentiation (bottom, CD11b as marker, day14) upon *Mbd6* KD. **e**,**f**, Spike-in calibrated ATAC-seq signals in WT (shNC WT) and *Tet2*-KO (shNC *Tet2* KO) HSPCs, as well as in *Mbd6*-KD (shMbd6 Tet2 KO) *Tet2*-KO HSPCs. Overall levels (**e**) and metagene profiles (**f**) were shown. **g**, Volcano plot comparing spike-in calibrated ATAC-seq peak signals in *Mbd6*-KD *versus* control *Tet2*-KO HSPCs. **h**,**i** Spike-in calibrated H2AK119ub levels in WT (shNC WT) and *Tet2*-KO (shNC *Tet2* KO) HSPCs, as well as in *Mbd6*-KD (shMbd6 Tet2 KO) *Tet2*-KO HSPCs. Overall levels (**h**) and metagene profiles (**i**) were shown. **j**, Volcano plot comparing spike-in calibrated H2AK119ub peak signals in *Mbd6* KD compared with control *Tet2*-KO HSPCs. **k**, Quantifications of in vitro differentiation and maintenance of HSPCs with *Nsun2*, *Nsun5* or *Trdmt1* KD. **l**, qPCR results assessing efficacies of shRNA-enabled *Nsun2*, *Nsun5* or *Trdmt1* KD in HSPCs. **m**, Half life measurements of IAP RNA in HSPCs of different genotypes. WT denotes wild-type, *Tet2* KO denotes *Tet2* KO, EE mutant lost hm^5^C oxidation activities and HxD mutant is catalytically inactive. Bar (**b**,**e**,**h**,**l**,**m**) or line centre (**k**,**m**) represents mean and whiskers s.d. (**b**,**k**-**m**) or s.e.m. (**e**,**h**). *P* values, Wald test (**g**,**j**), two-tailed unpaired *t* test with Welch’s correction (**b**,**e**,**h**,**k**-**m**). NS, *P* > 0.05. *n* = 3 biological replicates (**a**-**c**,**e**-**m**). The depicted genome-wide data represent an integration of three biological replicates. Source Data

*TET2* deficiency in haematopoietic stem and progenitor cells (HSPCs) is well known to cause open chromatin and genome instability, finally leading to myeloid malignancy^41^. The most important feature of TET2-deficient (*Tet2*^*−/−*^) HSPCs (Lin^−^KIT^+^ cells, capturing HSPCs) is an enhanced self-renewal capacity of haematopoietic stem cells and skewed differentiation towards granulocytic/monocytic lineages in vitro^4^. Consistent with our observations in mES cells, we observed a global increase in chromatin accessibility in TET2-deficient HSPCs (Extended Data Fig. 7a). We designed a chimera assay to study effects of *Mbd6* KD in vivo (Extended Data Fig. 7b), and performed competitive transplantation assays using WT + control short hairpin RNA (shNC), WT + shMbd6, *Tet2*^*−/−*^ + shNC or *Tet2*^*−/−*^ + shMbd6 CD45.2^+^ HSPCs versus CD45.1 competitor bone marrow (BM) cells (ratio of HSPCs at around 1:9; Fig. 4a). The donor cell (CD45.2^+^) chimerism in the recipients transplanted with *Tet2*^*−/−*^ + shNC HSPCs steadily increased, reaching around 50% at 6 months after transplantation (Fig. 4b), while the donor cell population in the peripheral blood (PB) of mice receiving *Tet2* KO + shMbd6 HSPCs remained comparable to those receiving WT/shNC or WT/shMbd6 HSPCs at a low level around 5% (Fig. 4b). Similar trends for the donor cell (CD45.2^+^) chimerism were observed in cells from the BM or spleen at 6 months after transplantation (Fig. 4c). Consistently, the recipients transplanted with *Tet2*^*−/−*^ + shNC HSPCs exhibited mild splenomegaly, while the spleen sizes from animals receiving WT + shNC, WT + shMbd6 or *Tet2*^*−/−*^ + shMbd6 HSPCs were normal (Fig. 4d,e), demonstrating almost full rescue of the defects caused by *Tet2* KO with additional *Mbd6* KD.

KD of *Mbd6* significantly reduced the replating potential of *Tet2*-KO HSPCs in vitro (Fig. 4f and Extended Data Fig. 7c). *Mbd6* KD disrupted the TET2-loss-induced prolonged maintenance of stem/progenitor cells and promoted differentiation of HSPCs towards myeloid lineages in vitro (Fig. 4g and Extended Data Fig. 7d). Consistently, *Mbd6* KD rescued global chromatin opening (Extended Data Fig. 7e–g) and H2AK119ub loss (Extended Data Fig. 7h–j) caused by *Tet2* KO, with the increased H2AK119ub negatively correlating with decreased chromatin accessibility in HSPCs (Fig. 4h). *Nsun2* KD partially rescued the prolonged maintenance and differentiation phenotypes of *Tet2*-KO HSPCs (Extended Data Fig. 7k,l). KD of other potential RNA m^5^C writer proteins (*Nsun5* and *Trdmt1*) did not alter these processes (Extended Data Fig. 7k,l). Similarly to that in mES cells, the IAP RNA lifetime was consistently elevated in *Tet2*-KO HSPCs, and these changes were dependent on the enzymatic activity of TET2 (Extended Data Fig. 7m). We further used a *Tet2* mutant that stalls TET2-mediated oxidation at the hm^5^C stage^42^. The IAP half-life profile of HSPCs from this mutant is similar to that of WT HSPCs, suggesting that potential hm^5^C oxidation by TET2 may not further contribute to IAP destabilization.

**Extended Data Fig. 8:**
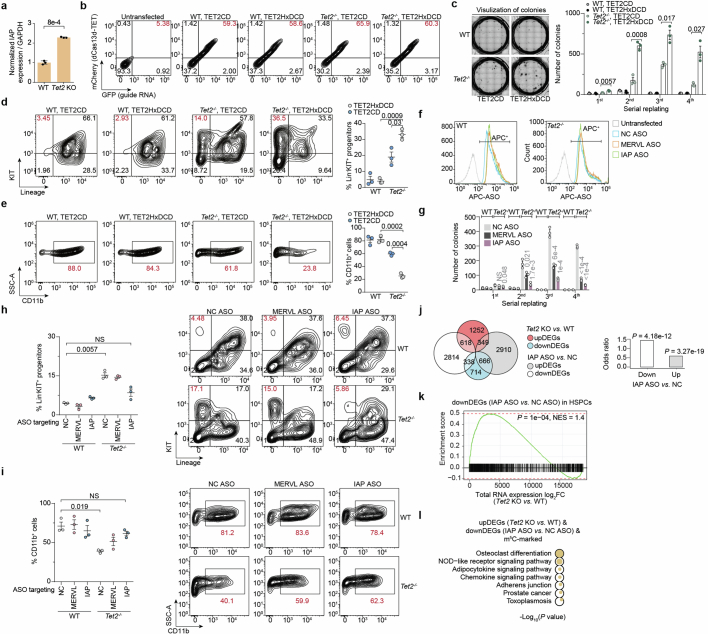
IAP is a key downstream carRNA target to rescue aberrant HSPC proliferation and differentiation caused by *Tet2* deficiency. **a**, RT-qPCR quantification for IAP levels in caRNAs isolated from WT or *Tet2*-KO (*Tet2*^−/−^) HSPCs. **b**, Representative flow cytometry analyses of dCas13d-TET2CD fusion protein and guide RNA transfection efficiency in HSPCs. **c**, Replating assay of HSPCs transfected with guide RNA targeting the IAP RNA using either the RfxdCas13d fusion protein with TET2 catalytic domain (TET2CD) or the inactivated mutant (TET2HxDCD). **d**, Flow cytometry analyses of HSPC frequency in suspension cultures (day 7) of WT and *Tet2*^*−/−*^ HSPCs transfected with guide RNA targeting the IAP RNA using the RfxdCas13d fusion protein with TET2CD or TET2HxDCD. **e**, Flow cytometry analyses of differentiation (CD11b, day 14) of WT and *Tet2*^*−/−*^ HSPCs transfected with guide RNA targeting the IAP RNA using the RfxdCas13d fusion protein with TET2CD or TET2HxDCD. **f**, Representative flow cytometry analyses of ASO transfection efficiency in HSPCs. **g**, Replating assay of WT and *Tet2*^*−/−*^ HSPCs transfected with control (NC ASO), MERVL-targeting (MERVL ASO) or IAP-targeting (IAP ASO) steric antisense oligos. Comparisons against “NC ASO” group. **h**, Flow cytometry analyses in suspension cultures (day 7) of the HSPCs frequency of WT and *Tet2*^*−/−*^ HSPCs transfected with NC ASO, MERVL ASO or IAP ASO. **i**, Flow cytometry analyses of differentiation (CD11b, day 14) of WT and *Tet2*^*−/−*^ HSPCs transfected with NC ASO, MERVL ASO or IAP ASO. **j**, Overlaps among differentially expressed genes upon *Tet2* KO or the treatment with IAP ASO in HSPCs (total RNA-seq). Barplot depicting the odds ratio of upDEGs following *Tet2* KO, in comparison to both upDEGs and downDEGs after treatment with IAP ASO in HSPCs. **k**, GSEA enrichment analysis conducted using genes downregulated (downDEGs) upon the treatment of ASO in HSPCs as the gene list and expression log_2_FC upon *Tet2* KO in HSPC cells as the rank list. **l**, Functional enrichment analysis on genes that are upregulated following *Tet2* KO and downregulated upon treatment with an ASO targeting IAP. These genes were located near m^5^C peaks. Bar (**a**,**c**,**g**) or line centre (**d**,**e**,**h**,**i**) represents mean and whiskers s.d. *P* values, two-tailed Fisher’s Exact Test (**j**), two-tailed unpaired *t* test with Welch’s correction (**a**,**c**-**e**,**g**-**i**), two-tailed permutation (**k**), one-tailed Fisher’s Exact Test (**l**). NS, *P* > 0.05. *n* = 3 biological replicates (**a**,**c**-**e**,**g**-**i**). The depicted genome-wide data represent an integration of three biological replicates. Source Data

Increased IAP abundance in *Tet2*-KO HSPC caRNA was also confirmed (Extended Data Fig. 8a). We proceeded to examine the functional outcomes of targeted IAP RNA m^5^C oxidation using the dCas13–TET2-CD fusion construct in HSPCs (Extended Data Fig. 8b). Targeted IAP m^5^C oxidation also partially rescued the enhanced replating potential of HSPCs by TET2 loss (Extended Data Fig. 8c). The expression level of stem/progenitor markers (Lin^−^KIT^+^; Extended Data Fig. 8d) and the myeloid lineage marker CD11b (Extended Data Fig. 8e) could be restored by targeted oxidation of IAP RNA m^5^C by ectopic expression of a dCas13–TET2-CD fusion protein, but not by its catalytic dead mutant in *Tet2-*KO HSPCs.

Similarly, steric blockade of m^5^C sites of IAP RNA partially rescued the enhanced replating potential of HSPCs by TET2 loss (Extended Data Fig. 8f,g). IAP blockade disrupted the prolonged maintenance of stem/progenitor cells and enhanced differentiation of HSPCs towards myeloid lineages (CD11b^+^) in vitro (Extended Data Fig. 8h,i).

To reveal the underlying mechanism, we conducted RNA-sequencing (RNA-seq) analysis of the effects of IAP ASO treatment. Genes downregulated, but not upregulated, by IAP ASO treatment exhibited a higher overlap with the genes upregulated by *Tet2* KO (Extended Data Fig. 8j,k). Further functional analysis of genes that were upregulated by *Tet2* KO, downregulated by IAP ASO treatment, and with nearby m^5^C-marked caRNA revealed enrichments in pathways including osteoclast differentiation and the NOD-like receptor signalling pathway (Extended Data Fig. 8l). Among these, SOCS3 acts as a potent inhibitor of HSPC differentiation^43^, while *Nfkbia* encodes a member of the NF-κB inhibitor family, therefore promoting HSPC proliferation^44,45^. Correspondingly, the chromatin accessibility at their genomic regions increased in *Tet2*-KO HSPCs but decreased after *Mbd6* KD (Fig. 4i).

## Targeting MBD6 in *TET2*-mutant leukaemia

**Fig. 5:**
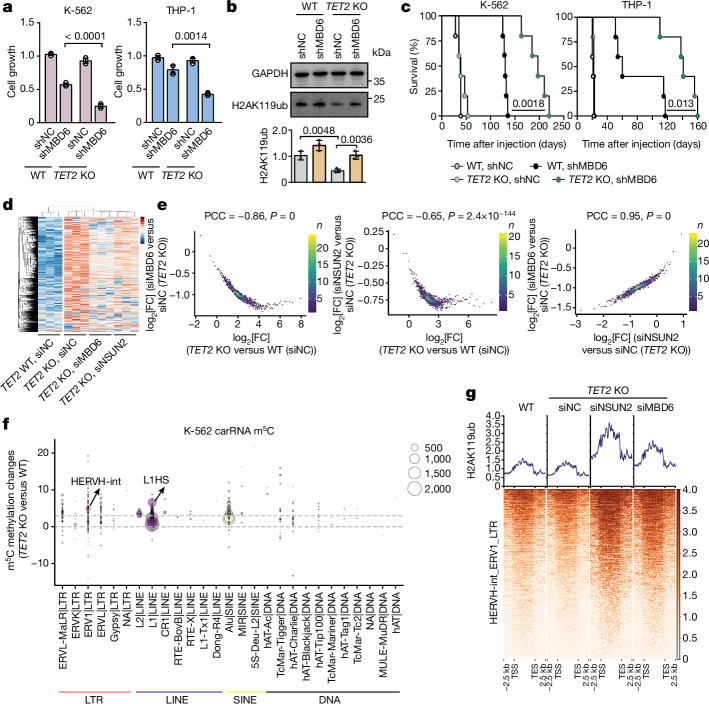
MBD6 exhibited synergistic effects with TET2 deficiency in regulating leukaemia progression through the m^5^C–H2AK119ub axis. **a**, Proliferation of WT or *TET2*-KO K-562 or THP-1 cells with (shMBD6) or without (shNC) *MBD6* KD. *n* = 4. **b**, The K-562 H2AK119ub level changes after *MBD6* KD. *n* = 3. **c**, NSG mice were transplanted with K-562 (left) or THP-1 (right) cells and their overall survival is shown as the Kaplan–Meier estimator. *n* = 5 mice. **d**, Heat map illustrating the spike-in-calibrated ATAC–seq signals on ATAC–seq peak regions in WT (siNC WT) and *TET2*-KO (siNC *TET2* KO) K-562 cells, as well as in *NSUN2* KD (siNSUN2 *TET2* KO) and *MBD6*-KD (siMBD6 *TET2* KO) *TET2*-KO K-562 cells. *n* = 3. Data are row-normalized using *z* scores. **e**, The correlation of changes in ATAC signals between *TET2-*KO versus WT K-562 cells, and comparing *NSUN2* KD or *MBD6* KD with control in *TET2-*KO K-562 cells. **f**, m^5^C methylation level changes of K-562 cells in different repeat RNA families after *TET2* KO. The size of the circle represents the number of loci methylated by m^5^C. **g**, The H2AK119ub signals around HERVH-int genomic loci. The colour bar shows H2AK119ub signal. Data are mean ± s.d. *P* values were calculated using two-tailed unpaired *t*-tests with Welch’s correction (**a** and **b**), log-rank Mentel–Cox tests (**c**) and two-tailed *t*-distribution with *n* − 2 d.f. (**e**). The depicted genome-wide data represent an integration of three biological replicates. Source Data

**Extended Data Fig. 9:**
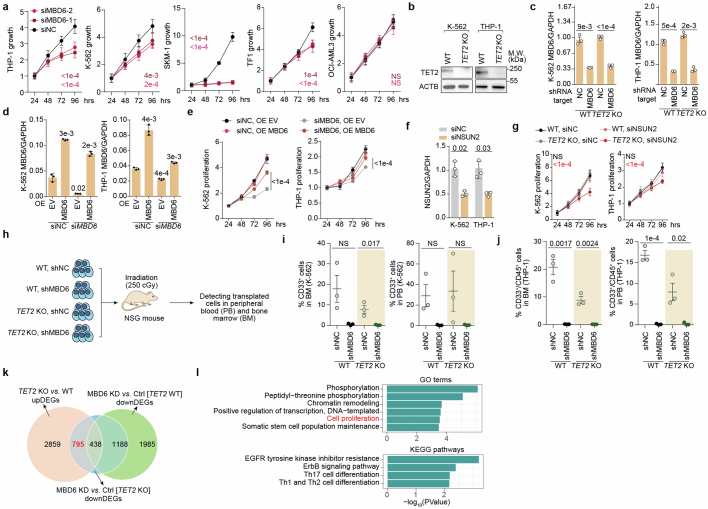
MBD6 is a targetable dependency in *TET2*-mutant leukaemia cells. **a**, Proliferation of human leukaemic cell lines upon *MBD6* KD using two individual siRNAs. Comparisons against “siNC” group at 96 h. **b**, Western blot analysis of TET2 levels in WT and *TET2*-KO K-562 and THP-1 cells. **c**, qPCR results validating efficiency of shRNA-enabled *MBD6* KD in K-562 or THP-1 cells. **d**, Validation of *MBD6* KD and rescue via qPCR analysis. **e**, Proliferation curves showing that overexpressing MBD6 could rescue growth arrest caused by *MBD6* KD. Comparisons against “si*MBD6* OE EV” group at 96 h. **f**, RT-qPCR validation of *NSUN2* KD in K-562 and THP-1 cells. **g**, Proliferation curves of K-562 and THP-1 cells upon *NSUN2* KD. Comparisons against “siNC” groups of WT or *TET2*-KO, individually. **h**, NSG mice were intravenously injected with K-562 or THP-1 cells as indicated and their overall survival is depicted in the Kaplan-Meier estimator (*n* = 5 mice). **i**, Fractions of CD33^+^ cells in BM or PB isolated from NSG mice xenotransplanted with tumour cells (K-562) for 28–39 days. **j**, Fractions of CD33^+^/CD45^+^ cells in BM or PB isolated from NSG mice xenotransplanted with tumour cells (THP-1) for 20–23 days. **k**, Venn diagram showing genes upregulated following *TET2* KO, genes downregulated upon *MBD6* KD in WT K-562, and genes downregulated upon *MBD6* KD in *TET2*-KO K-562 cells. **l**, Functional enrichment analysis of genes that respond to both *TET2* KO, and subsequent *MBD6* KD in K-562 cells, but not to *MBD6* KD alone in WT K-562 cells. Bar (**c**,**d**,**f**) or line centre (**a**,**e**,**g**,**i**,**j**) represents mean and whiskers s.d. *P* values, two-tailed unpaired *t* test with Welch’s correction (**a**,**c**-**g**,**i**,**j**), one-tailed Fisher’s Exact Test (**l**). NS, *P* > 0.05. Biological replicates, *n* = 6 (**a**), *n* = 4 (**g**), *n* = 3 (**b**-**d**,**f**,**i**,**j**). The depicted genome-wide data represent an integration of three biological replicates. Source Data

After we demonstrated that the m^5^C–TET2–LTR–MBD6 axis is important for HSPC function, we next studied its role in leukaemia fitness. While modest inhibition of proliferation was observed in *TET2* WT cell lines, almost complete proliferation blockade was observed for SKM-1 cells, an acute myeloid leukaemia (AML) cell line bearing a *TET2* mutation, after *MBD6* KD (Extended Data Fig. 9a). To further confirm this synergistic lethal effect, we compared the proliferation of WT, *TET2-*KO K-562 and *TET2*-KO THP-1 cells with *MBD6* depletion (Extended Data Fig. 9b,c). *MBD6* KD markedly attenuated proliferation of *TET2-*KO cells (Fig. 5a), along with an increased global H2AK119ub levels (Fig. 5b). The attenuated growth of *TET2*-KO K-562 and THP-1 cells by *MBD6* KD could be rescued by *MBD6* overexpression (Extended Data Fig. 9d,e). We also observed synergistic inhibition of proliferation when knocking down *NSUN2* in *TET2*-KO K-562 and THP-1 cells (Extended Data Fig. 9f,g).

To test whether MBD6 loss affects leukemogenesis in vivo, especially in the absence of TET2, we transplanted WT + shNC, *TET2* KO + shNC, WT + shMBD6 or *TET2*-KO + shMBD6 K-562 cells into adult *NOD.Cg-Prkdc*^*scid*^*Il2rg*^*tm1Wjl*^*/SzJ* (NSG) mice (Extended Data Fig. 9h). Mice receiving shMBD6 cells exhibited substantially decelerated leukemogenesis compared with shNC cells; those that were transplanted with *TET2* KO + shMBD6 cells survived significantly longer (125–135 days or 163–220 days, respectively) (Fig. 5c). Consistently, shNC-recipient mice showed markedly higher human chimerism in the BM and PB than the shMBD6-recipient mice (Extended Data Fig. 9i). Similar results were also observed in an in vivo xenotransplantation study with THP-1 cells under the same experimental settings (Fig. 5c). *MBD6* KD prolonged survival significantly longer in mice receiving *TET2* KO + shMBD6 cells than WT + shMBD6 cells. WT or *TET2*-KO recipient mice showed substantially higher human CD33^+^CD45^+^ cell chimerism in the BM and PB compared with WT + shMBD6 or *TET2* KO + shMBD6 recipient mice (Extended Data Fig. 9j). Thus, *MBD6* KD markedly attenuated leukaemic progression in vivo, specifically in the absence of TET2.

*MBD6* KD could also suppress transplanted *TET2* WT human cells in vivo (Extended Data Fig. 9i,j). We hypothesized that, in *TET2* WT cells, MBD6 maintains the expression of pro-proliferating genes, with a larger portion suppressed by TET2. TET2 depletion led to the activation of these genes, with malignant cells becoming addictive to these pathways for proliferation. Supporting our hypothesis, we found that genes that were specifically downregulated by *MBD6* KD in *TET2-*KO cells, rather than in WT controls, were enriched with cell proliferation (Extended Data Fig. 9k,l).

**Extended Data Fig. 10:**
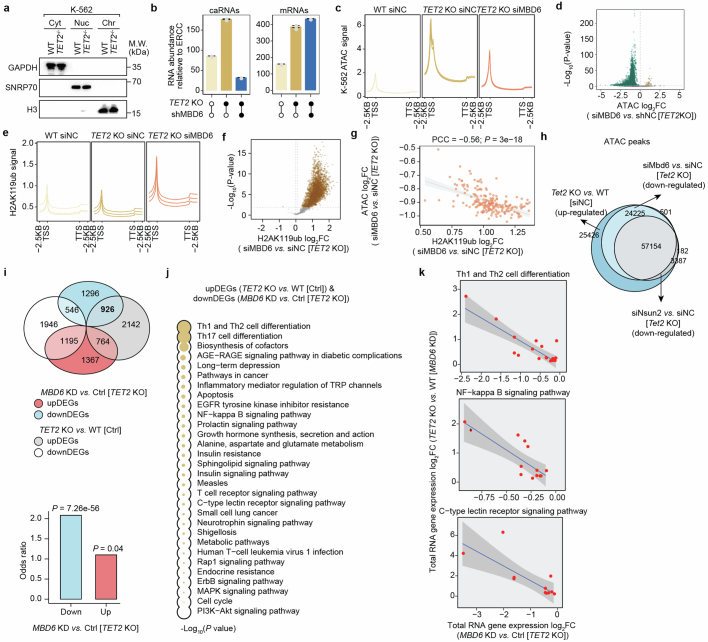
MBD6 governs a transcription program in leukaemic cell through regulating H2AK119ub. **a**, Representative subcellular fractionation of K-562 cells. Cyt: cytosolic; Nuc: nuclear soluble; Chr: chromatin. **b**, Overall caRNA and whole-cell RNA abundance relative to spike-in in WT, *TET2*-KO and *TET2*-KO + *MBD6*-KD K-562 cells. **c**, Metagene profile of the calibrated ATAC-seq signals of WT (siNC WT) and *Tet2* KO (siNC *Tet2* KO) K-562 cells, as well as in *Mbd6* KD (siMbd6 Tet2 KO) *Tet2* KO K-562 cells. **d**, Volcano plot comparing spike-in calibrated ATAC-seq peaks in *Mbd6*-KD *versus* control in *Tet2*-KO K-562 cells. **e**, Metagene profile of the spike-in calibrated H2AK119ub signals of WT (siNC WT) and *Tet2*-KO (siNC *Tet2* KO) K-562 cells, as well as in *Mbd6*-KD (siMbd6 Tet2-KO) *Tet2*-KO K-562 cells. **f**, Volcano plot comparing spike-in calibrated H2AK119ub peaks in *Mbd6*-KD compared with control *Tet2*-KO K-562 cells. **g**, Correlation between changes in ATAC and H2AK119ub signals, comparing *Mbd6*-KD with control in *Tet2*-KO K-562 cells. **h**, Venn diagram displaying the significantly up-regulated ATAC-seq peaks in *Tet2*-KO compared with WT K-562 cells, and down-regulated ATAC-seq peaks in *Nsun2*-KD or *Mbd6*-KD compared with control *Tet2*-KO K-562 cells. **i**, Overlap of differentially expressed genes upon *TET2* KO in control K-562 cells, and upon *MBD6* KD in *TET2*-KO K-562 cells. Barplot depicting the odds ratio of upDEGs following *TET2* KO, in comparison to both upDEGs and downDEGs upon *MBD6* KD in *TET2* KO K-562 cells. **j**, KEGG pathway enrichment analysis performed on genes that were upregulated in *TET2*-KO in control K-562 cells, and downregulated in *MBD6*-KD in *TET2*-KO K-562 cells. Larger circles indicate greater significance. **k**, Correlation of gene expression changes between *TET2* KO *versus* WT, and MBD6 KD *versus* control in *TET2* KO K-562 cells. The genes are associated with various signalling pathways, including Th1 and Th2 cell differentiation, NF-kappa B signalling pathway, and C-type lectin receptor signalling pathways. Error band represents standard errors. Bar represents mean (**b**) and whiskers s.e.m. *P* values, Wald test (**d**,**f**), two-tailed Fisher’s Exact Test (**i**), one-sided Fisher’s Exact test (**j**), two-tailed t-distribution with n-2 degrees of freedom (**g**). *n* = 3 biological replicates (**a**-**c**,**e**). The depicted genome-wide data represent an integration of three biological replicates.

*MBD6* KD reversed the excessive caRNA expression after *TET2* KO, but not for whole-cell RNA in K-562 cells (Extended Data Fig. 10a,b). Consistent with what we observed in mES cells, *MBD6* KD was able to rescue the more-open chromatin state (Extended Data Fig. 10c,d) and decreased H2AK119ub (Extended Data Fig. 10e,f) caused by *TET2* KO. The increases in H2AK119ub correlate well with the decreases in ATAC signals (Extended Data Fig. 10g). Examination of chromatin changes caused by *NSUN2* KD corroborated the dependence of this regulation on RNA m^5^C (Fig. 5d,e and Extended Data Fig. 10h).

LTR, particularly the ERV1 family with HERVH-int as representative subfamilies, exhibited higher m^5^C methylation changes in *TET2-*KO K-562 cells (Fig. 5f). MBD6 and NSUN2 also similarly regulate the H2AK119ub levels on HERVH-int elements (Fig. 5g). Finally, we investigated the downstream signalling pathways that are involved in this m^5^C–TET2–MBD6–BAP1 axis in leukaemia cells. We found a notable overlap between the genes upregulated by *TET2* KO and those downregulated by *MBD6* KD in *TET2*-depleted K-562 cells (Extended Data Fig. 10i). The enriched terms of these overlapped genes are highly conserved, similar to what we observed in *Tet2* KO and IAP ASO treatment in mouse HSPCs (Extended Data Fig. 10j,k).

## Discussion

carRNAs offer a platform for dynamic chromatin regulation. Recent studies have shown that caRNA *N*^6^-methyladenosine modification has essential roles in global and local chromatin state regulation during mouse early embryo development and in the progression of cancer^21–25,46^, with the methyltransferase METTL3 functioning as a writer, the binding proteins such as YTHDC1 and RBFOX2 functioning as readers, and FTO functioning as an eraser to reversibly control transcription. We speculated that other RNA modifications may be present on carRNAs and affect chromatin regulation in a similar manner.

TET2 can mediate DNA 5mC oxidation and is an established tumour suppressor for myeloid malignancies. However, *TET2* mutations are known to cause global DNA hypomethylation instead of hypermethylation (if it functions as a DNA demethylase), a puzzle that lacks mechanistic explanation. By analysing genomic features associated with hypomethylated or hypermethylated genomic regions, we observed that DNA hypermethylation occurs to enhancers but hypomethylation occurs to repetitive elements, with hypermethylated regions overlapping well with CXXC5-bound genomic loci while hypomethylated regions are enriched in BAP1 binding. We further identified that TET2 mediates RNA m^5^C methylation on carRNA, in particular the LTR repeat RNA, to regulate chromatin state and transcription. A very recent report described caRNA m^5^C oxidation in glioma^47^, but the connection between m^5^C and its oxidation with chromatin state change was not established. We found that MBD6 preferentially recognizes m^5^C on the repeat RNA, which recruits the BAP1 complex to mediate H2AK119ub deubiquitination and gene activation. The m^5^C oxidation by TET2 on these LTR RNAs antagonizes gene activation through the m^5^C–MBD6–BAP1 deubiquitination axis. Loss of TET2 leads to caRNA m^5^C hypermethylation, more-open chromatin and widespread DNA hypomethylation that activates genes critical for leukaemogenesis, explaining the accelerated myeloid malignancy induced by TET2 inactivation (Fig. 4i and Extended Data Fig. 8l).

Our studies also suggest a bimodal function of TET2—when TET2 binds to CXXC4/CXXC5, it mediates DNA 5mC oxidation at the enhancer; however, when recruited by RNA-binding proteins such as PSPC1, TET2 mediates chromatin-associated repeat RNA m^5^C oxidation; this RNA m^5^C oxidation activity by TET2 dictates the global chromatin regulation in mES cells, HPSCs and leukaemia cells. This study therefore reveals a NSUN2–TET2–MBD6–BAP1 axis in chromatin and transcription regulation through repeat RNA m^5^C. Practically, it provides targets for future targeted therapies against *TET2* mutant malignancies.

## Methods

### Animals and tissues

*Tet2*^*−/−*^ mice were generated as described^41^. These mice used in this study were backcrossed for more than six generations with C57BL/6 mice. WT C57BL/6 and *Tet2*^*−/−*^ mice (aged 6–8 weeks), including both male and female, were used throughout this study and maintained under standard laboratory housing conditions with food and water ad libitum. All the mice were randomly assigned to experimental groups and data analyses were blindly performed by two lab members independently. All animal studies were performed with the approval from the Institutional Animal Care and Use Committee (IACUC), protocol number 30979/20190086AR at The University of Texas Health Science Center at San Antonio (UTHSCSA) and conducted in accordance with the institutional and national guidelines and regulations.

### Xenotransplantation of human leukaemia cells

For in vivo xenotransplantation study procedures, 1 × 10^6^ K-562 cells were injected intravenously via the tail vein into adult NSG mice (aged 6–8 weeks) pretreated with 250 cGy whole body irradiation. At 28–39 days after transplantation, PB was collected from the submandibular vein, and the BM was isolated from the tibias and femurs. Human CD33^+^ chimerism in BM and PB cells were analysed by BD FACSCelesta flow cytometer (BD Biosciences).

2 × 10^4^ THP-1 cells were injected intravenously through the tail vein into adult NSG mice (6–8 weeks old) pretreated with 250 cGy whole-body irradiation. At 20–22 days after transplantation, human CD33^+^CD45^+^ chimerism in BM and PB cells were analysed using the BD FACSCelesta flow cytometer.

A cohort of mice from each transplantation group was monitored until they became moribund or died.

### Competitive repopulation assay

The competitive repopulation assay was performed to assess the effect of *TET2* and/or *MBD6* KD on the repopulating potential of HSPCs in vivo. In total, 2 × 10^4^ Lin^−^KIT^+^ cells isolated from the BM cells of 8-week-old WT or *Tet2-*KO mice (CD45.2^+^) were lentivirally transduced with short hairpin RNA (shRNA) plasmid pLKO.1-shC002 (MilliporeSigma, SHC002: shNC) or pLKO.1-sh*Mbd6* (Millipore-Sigma, TRCN0000178563) and incubated in suspension culture containing 20% FBS in complete RPMI-1640 medium supplemented with 100 ng ml^−1^ mSCF, 10 ng ml^−1^ mIL-3, 10 ng ml^−1^ IL-6 and 20 ng ml^−1^ mFlt3. Then, 48 h after transduction, Lin^−^KIT^+^ cells from each transduction were transplanted along with 1 × 10^6^ 8-week-old BoyJ (CD45.1^+^) BM competitor cells into lethally irradiated (800 cGy) BoyJ recipients through the tail-vein injection. The CD45.2/CD45.1 chimeras in the PB were monitored monthly for 6 months. Recipients were euthanized 6 months after transplantation to analyse the CD45.2/CD45.1 chimeras in the BM and spleen.

### Haematopoietic stem and progenitor cell sorting, colony assay and in vitro differentiation assay

For haematopoietic stem and progenitor Lin^−^KIT^+^ cell selection, magnetic-activated cell sorting was applied with autoMACS Pro Separator (Miltenyi Biotec). In brief, the lineage-positive cells (Lin^+^) were depleted from total BM cells of 6–8-week-old mice using the Direct Lineage Cell Depletion Kit (Miltenyi Biotec, 130-110-470), and the Lin^−^ cells were then sorted with KIT (CD117) MicroBeads (Miltenyi Biotec, 130-091-224). The purity of selected cells was analysed by flow cytometry.

For colony assay, HSPCs were plated in triplicate in methylcellulose medium (MethoCult, M3134) supplemented with mouse stem cell factor (mSCF; 100 ng ml^−1^), interleukin-3 (mIL-3; 10 ng ml^−1^), thrombopoietin (mTPO; 50 ng ml^−1^), granulocyte-macrophage colony-stimulating factor (mGM-CSF; 10 ng ml^−1^), human erythropoietin (hEPO; 4 U ml^−1^) and interleukin-6 (hIL-6; 50 ng ml^−1^, PeproTech). The colonies were imaged using STEMvision (StemCell Technologies) and scored on day 7, and these colonies were then sequentially replated every 7 days for replating assay. Colony cells were also collected and analysed for expression of stem and progenitor markers and myeloid linage markers by flow cytometry.

The HSPCs were also incubated in suspension culture containing 30% FBS and 2% BSA in complete RPMI-1640 medium supplemented with 100 ng ml^−1^ mSCF, 10 ng ml^−1^ mIL-3, 50 ng ml^−1^ mTPO and 10 ng ml^−1^ mGM-CSF. Cells were collected and analysed for expression of stem/progenitor markers at day 7 and myeloid lineage markers at day 14 by flow cytometry.

### Flow cytometry analysis

Cells were stained with PerCP-Cy5.5 mouse lineage antibody cocktail (BD Biosciences, 561317) and PE rat anti-mouse CD117 (BD Biosciences, 553869) antibody for haematopoietic stem and progenitor cells analysis. Brilliant Violet 421 (BV421) anti-mouse/human CD11b (Mac-1) (BioLegend, 101236) was used to analyse myeloid lineage. PerCP-Cy5.5 mouse anti-mouse CD45.2 (BD Biosciences, 552950) and FITC mouse anti-mouse CD45.1 (BD Biosciences, 553775) antibodies were used for analysing CD45.2/CD45.1 chimeras in a competitive repopulation assay.

Human CD33 chimerism was analysed with PE mouse anti-human CD33 (BD Biosciences, 561816) and PE-Cy7 rat anti-mouse CD45 (BD Biosciences, 552848) in PB and BM cells from NSG mice that were xenotransplanted with K-562 cells. Human CD33/CD45 chimerism was analysed with PE mouse anti-human CD33 (BD Biosciences, 561816) and APC mouse anti-human CD45 (BD Biosciences, 555485) in PB and BM cells from NSG mice that were xenotransplanted with THP-1 cells. All flow cytometry data were analysed using FlowJo-V10 software (TreeStar). Examples of the gating strategies are provided in Supplementary Figs. 2 and 3.

### Cell culture

WT and *Tet2*^*−/−*^ mES cells were gifts from the B. Ren laboratory^26,48^. The control and KO mES cells have been shown to be pluripotent by chimera formation assay. All mES cells were kept in DMEM (Gibco, 11995065) supplemented with 15% heat-inactivated stem-cell-qualified fetal bovine serum (Gemini Bio Products, 100-525), 1× l-glutamine (Gibco, 25030081), NEAA (Gibco, 25030081), LIF (Millipore-Sigma, ESG1107), 1× β-mercaptoethanol (Gibco, 21985023), 3 μM CHIR99021 (StemCell Technologies, 72052) and 1 μM PD0325901 (StemCell Technologies, 72182) at 37 °C and 5% CO_2_. For stable TET2 overexpression mES cells, empty vector, WT *Tet2* or *Tet2* HxD mutant bearing piggyBac plasmids were constructed and transfected into *Tet2*-KO or *Pspc1-*KO mES cells using Lipofectamine 3000 Transfection Reagent (Invitrogen, L3000001) according to the standard protocol. Stable expression clone selection was performed using 0.1 mg ml^−1^ hygromycin B (Gibco, 10687-010) for 2 weeks. The medium was replaced every 24 h. ES cells were passaged on gelatin-coated plates twice to clear feeder cells before experiments.

WT THP-1, K-562 and TF-1 cells were obtained from the American Type Culture Collection (ATCC). The SKM-1 cell line was obtained from DSMZ (German Collection of Microorganisms and Cell Cultures). WT OCI-AML3 cell was a gift from L. Godley. WT and *TET2*^*−/−*^ K-562 and THP-1 cells were gifts from B. K. Jha as previously generated^49^. THP-1, K-562, SKM-1 and OCI-AML3 cells were kept in RPMI-1640 (Gibco, 61870036) with 10% fetal bovine serum (FBS, Gibco 26140079) at 37 °C under 5% CO_2_. TF-1 was kept in RPMI-1640 (Gibco, 61870036) with 10% FBS (Gibco 26140079) and 2 ng ml^−1^ recombinant GM-CSF (Peprotech, 300-03) at 37 °C under 5% CO_2_. U-87 MG (HTB-14), LN-229 (CRL-2611), Hep G2 (HB-8065), HeLa (CCL-2), HCT 116 (CCL-247), A549 (CCL-185) and A-375 (CRL-1619) cells were obtained from the American Type Culture Collection (ATCC). U-87 MG and LN-229 were kept in ATCC-formulated Eagle’s minimum essential medium (ATCC, 30-2003) supplemented with 10% FBS (Gibco, 26140079) and 5% FBS (Gibco, 26140079), respectively. Hep G2, HeLa, HCT 116, A549 and A-375 cells were kept in DMEM (Gibco, 11995065) supplemented with 10% FBS (Gibco, 26140079). All cell types were kept at 37 °C and 5% CO_2_.

shNC and shMBD6 THP-1 and K-562 cell lines were constructed by lentivirus transduction with TransDux MAX Lentivirus Transduction Reagent (System Biosciences, LV860A-1). Lentiviral particles were prepared by using HEK293T cells and lentiviral packaging plasmids pCMV-VSV-G and pCMV-dR8.2 (pCMV-VSV-G and pCMV-dR8.2 were gifts from B. Weinberg (Addgene plasmid, 8454; and Addgene plasmid, 8455)) and shRNA plasmid pLKO.1-shC002 (Millipore-Sigma, SHC002) or pLKO.1-shMBD6 (Millipore-Sigma, TRCN000038787). Then, 48 h after transfection, lentiviral particles were precipitated using the PEG-it Virus Precipitation Solution (System Biosciences, LV810-1). shNC and shMBD6 THP-1 and K-562 cell lines were kept in RPMI-1640 (Gibco, 61870036) with 10% fetal bovine serum (FBS, Gibco) and 1 μg ml^−1^ puromycin (Gibco, A1113803) at 37 °C under 5% CO_2_. Small interfering RNA (siRNA) or gene overexpression plasmids transfection in K-562 and THP-1 cells were performed according to the manufacturer’s instructions for SF Cell Line 4D-Nucleofector X Kit (Lonza Biosciences, V4XC-2032, FF-120 for K-562) or SG Cell Line 4D-Nucleofector X Kit (Lonza Biosciences, V4XC-3024, FF-100 for THP-1)

*TET2*-KO THP-1 cell line for PDX model was generated using CRISPR–Cas9 system. Single-guide RNAs were designed using the CRISPick tool (https://portals.broadinstitute.org/gppx/crispick/public) and then cloned into LentiCRISPR V2-GFP vector by Synbio Technologies. THP-1 cells were infected by lentiviral particles for 72 h and followed by GFP-positive cell selection using the BD FACSMelody Cell Sorter (BD Biosciences). KO efficiency was verified by western blotting.

shNC, shMBD6 (Millipore-Sigma, TRCN0000178563), shNsun2 (Millipore-Sigma, TRCN0000325347), shNsun5 (Millipore-Sigma, TRCN0000097512) or shTrdmt1 (Millipore-Sigma, TRCN0000328293) Lin^−^KIT^+^ HSPCs were constructed by electroporation with the P3 Primary Cell 4D-Nucleofector X Kit S (Lonza Bioscience, V4XP-3032) by program CV-137.

### siRNA and plasmid transfection

Two or three individual siRNAs, or a pool of four siRNAs targeting different regions of the same transcript (Dharmacon siRNA) were used for KD of human or mouse transcripts. siRNA transfections in mES cells and other adherent cell lines were performed using Lipofectamine RNAiMAX Transfection Reagent (Invitrogen, 13778075) according to the manufacturer’s instructions. Transfections in human leukaemia cells (THP-1, TF-1, OCI-AML3, SKM-1) were performed by electroporation using the SG Cell Line 4D-Nucleofector X Kit L (Lonza Bioscience, V4XC-3024) with program FF-100. Transfections in K-562 cells were performed with the SF Cell Line 4D-Nucleofector X Kit L (Lonza Bioscience, V4XC-2012) with program FF-120.

Plasmid transfections in mES cells or HEK293T cells were performed using the Lipofectamine 3000 Transfection Reagent (Invitrogen, L3000015) according to the manufacturer’s instructions.

### Cell proliferation assay

The cell proliferation assays for adherent and suspension cells were performed similarly. Cells were seeded into 96-well plates before assaying in 100 μl settings with CellTiter 96 Aqueous One Solution Cell Proliferation Assay (Promega, G3582) according to the manufacturer’s instructions. Then, 2,000–10,000 cells were seeded per well at day 0 and the cell proliferation was monitored every 24 h by incubating the cell suspension with MTS reagent at 37 °C for 1 h.

### DNase I–TUNEL assay

For cell line samples, mES cells were reseeded to 10 cm cell culture dishes 12 h before siRNA transfection. The DNase I–TUNEL assay was performed using DeadEnd Fluorometric TUNEL System (Promega, G3250) according to the manufacturer’s instructions after cell fixation with paraformaldehyde and permeabilization with Triton X-100. Two independent experiments were performed. Cells were treated with 1 U ml^−1^ of DNase I (Thermo Fisher Scientific, EN0521) for 5 min at 37 °C before rTdT labelling. Flow cytometry was performed on a BD Fortessa (BD Biosciences), and data were analysed using Flowjo (TreeStar).

### Nascent RNA imaging assay

mES cells were reseeded in Nunc Lab-Tek II Chambered Coverglass (Thermo Fisher Scientific, 155409) 12 h before treatment. The nascent RNA synthesis assay was performed using Click-iT RNA Alexa Fluor 488 Imaging Kit (Invitrogen, C10329) according to the manufacturer’s instructions. 5-Ethynyl uridine incubation was performed for 1 h before washing away by cell medium. Cell nucleus was counterstained with Hoechst 33342 (Abcam, ab228551). The samples were imaged on a Leica SP8 laser scanning confocal microscope at University of Chicago. The fluorescence intensity across different samples were quantified with Cellprofiler v.3.0 with a custom workflow. The total RNA synthesis rate was obtained by multiplying the average intensity in each cell by the area of each cell.

### ATAC–see analysis

Assay of transposase-accessible chromatin with visualization (ATAC–see) of mES cells was performed as described in the original report^50^. ATTO-590-labelled imaging oligos were purchased from Integrated DNA Technologies (IDT) and the oligonucleotide sequences are as follows: Tn5MErev, 5′-[phos]CTGTCTCTTATACACATCT-3′; Tn5ME-A-ATTO590, 5′-/5ATTO590/TCGTCGGCAGCGTCAGATGTGTATAAGAGACAG-3′; Tn5ME-B-ATTO590: 5′-/ATTO590/GTCTCGTGGGCTCGGAGATGTGTATAAGAGACAG-3′. The oligos were assembled with recombinant Tn5 transposase (Active motif, 81286) to produce the Tn5 transposome. Cell fixation, permeabilization and labelling were performed as described in the original report^50^.

### Recombinant protein purification

Standard molecular cloning strategies were used to generate C-terminally MBP–6×His-tagged MBD domain of MBD6 (residues 1–100). The human *MBD6* coding sequence was obtained from Origene (Origene, SC324058). The full-length coding sequence was cloned using PrimeSTAR GXL DNA Polymerase (TaKaRa Bio, R050B). Recombinant proteins were expressed in *E. coli* BL21 (DE3) grown to an optical density at 600 nm of 0.6 in LB medium. The expression was induced with 0.6 mM IPTG at 16 °C for 20 h and cells were collected by centrifugation.

For purification of MBP tagged MBD domain of MBD6, bacterial pellet was resuspended in a lysis buffer containing 25 mM Tris-HCl (pH 7.5), 500 mM NaCl, 20 mM imidazole, 10 mM β-mercaptoethanol (β-ME) and protease inhibitors (ethylenediaminetetraacetic-acid-free protease inhibitor cocktail tablet, Millipore-Sigma 4693132001) and disrupted by sonication for 3 min. The cell lysates were clarified by centrifugation at 26,000*g* for 30 min and the supernatant was applied to Ni^2+^-NTA resin (Thermo Fisher Scientific, 88221) and washed with lysis buffer, and the bound proteins were eluted with lysis buffer supplemented with 250 mM imidazole. The eluted protein was bound back to amylose resin (NEB, E8021S) before washing with lysis buffer. The bound protein was eluted with 1% maltose in lysis buffer. The eluted protein was analysed by SDS–PAGE and concentrated by centrifugal filtration (Amicon Ultra-15). Final concentrated protein was aliquoted, flash-frozen and stored at −80 °C for future use.

### RT–qPCR

To quantify expression levels of transcripts, total RNA was reverse transcribed using the PrimeScript RT Master Mix (TaKaRa Bio, RR0361) with oligo dT primer and random hexamers as primers. The cDNA was then subjected to quantitative PCR (qPCR; LightCycler 96 system, Roche) using FastStart Essential DNA Green Master (Roche, 06402712001) with gene-specific primers. The relative changes in expression were calculated using the ΔΔ*C*_t_ method.

### Western blot analysis

Protein samples were prepared from respective cells by lysis in RIPA buffer (Thermo Fisher Scientific, 89900) containing 1× Halt protease and phosphatase inhibitor cocktail (Thermo Fisher Scientific 78441). Protein concentration was measured by NanoDrop 8000 Spectrophotometer (Thermo Fisher Scientific). Lysates of equal total protein concentration were heated at 90 °C in 1× loading buffer (Bio-Rad, 1610747) for 10 min. Denatured protein was loaded into 4–12% NuPAGE Bis-Tris gels (Invitrogen, NP0335BOX) and transferred to PVDF membranes (Thermo Fisher Scientific, 88585). Membranes were blocked in Tris-buffered saline, 0.1% Tween-20 (TBST) with 3% BSA (Millipore-Sigma, A7030) for 30 min at room temperature, incubated in a diluted primary antibody solution at 4 °C overnight, then washed and incubated in a dilution of secondary antibody conjugated to HRP for 1 h at room temperature. Protein bands were detected using SuperSignal West Dura Extended Duration Substrate kit (Thermo Fisher Scientific, 34075) with a FluroChem R (Proteinsimple). Blot intensities were quantified with Fiji (ImageJ) Analyse-Gel module. Uncropped gels with size marker indications are provided in Supplementary Fig. 1.

### Dot blot

Oligonucleotide probes end-labelled with Alexa Fluor 488 dye was spotted on a positively charged Nylon membrane (Roche, 11209299001). The membrane was dried at room temperature for 5 min before UV cross-linking at 254 nm with a Stratalinker (Stratagene) for two times to achieve a 4,500 J m^−2^ UV flux. The membrane was then blocked in Tris-buffered saline, 0.1% Tween-20 (TBST) with 3% BSA (Millipore-Sigma, A7030) for 30 min at room temperature. Primary antibodies were diluted according to the manufacturer’s instructions and incubated with the membrane for 60 min at room temperature. The membrane was washed and incubated in a dilution of secondary antibody conjugated to HRP for 60 min at room temperature. The final membrane was detected using SuperSignal West Dura Extended Duration Substrate kit (Thermo Fisher Scientific, 34075) with the iBright 1500 system (Invitrogen, A44241).

### Cell fractionation

Fractionation of mES cells, K-562 or THP-1 cells was performed according to the published protocol^51^ with the optimized concentration of NP-40 (MilliporeSigma, 492018) for each cell line. In brief, 5 × 10^6^ to 1 × 10^7^ cells were collected and washed with 1 ml cold PBS/1 mM EDTA buffer, then centrifuged at 4 °C and 500*g* to collect the cell pellet. Then, 200 μl ice-cold lysis buffer (10 mM Tris-HCl, pH 7.4, 0.05% NP-40, 150 mM NaCl) were added to the cell pellet and incubated on ice for 5 min, then gently pipetted up the cell lysate over 2.5 volumes of chilled sucrose cushion (24% RNase-free sucrose in lysis buffer) and centrifuged at 4 °C and 15,000*g* for 10 min. All the supernatant was collected as cytoplasmic fraction and the nuclei pellet was washed once by gently adding 200 μl ice-cold PBS/1 mM EDTA to the nuclei pellet without dislodging the pellet. The nuclei pellet was resuspended in 200 μl prechilled glycerol buffer (20 mM Tris-HCl, pH 7.4, 75 mM NaCl, 0.5 mM EDTA, 0.85 mM DTT, 0.125 mM PMSF, 50% glycerol) with gentle flicking of the tube. An equal volume of cold nucleus lysis buffer (10 mM HEPES, pH 7.6, 1 mM DTT, 7.5 mM MgCl_2_, 0.2 mM EDTA, 0.3 M NaCl, 1 M urea, 1% NP-40) was then added, followed by vigorous vertexing for 5 s twice. The nuclei pellet mixtures were incubated for 2 min on ice, then centrifuged at 4 °C and 15,000*g* for 2 min. The supernatant was collected as the soluble nuclear fraction (nucleoplasm). The pellet was gently rinsed with cold PBS/1 mM EDTA without dislodging and was then collected as the chromosome-associated fraction.

Fractionation of HSPCs was performed similar to ES cells with minor modifications. In brief, HSPCs were cultured in vitro for 2 h after sorting on the autoMACS Pro Separator, and then ice-cold lysis buffer (10 mM Tris-HCl, pH 7.4, 0.15% IGEPAL CA-630, 75 mM NaCl) was used to separate the cytoplasmic fraction. The procedures for isolating the nuclear fraction and chromosome-associated fraction were the same as that of ES cells.

### Quantitative analysis of modified base levels using UHPLC–MS/MS

The nucleic acid digestion step for RNA was as follows: 75 ng ribo-depleted RNA was digested by nuclease P1 (MilliporeSigma, N8630) in 20 μl buffer containing 20 mM ammonium acetate at pH 5.3 for 2 h at 42 °C. Then, 1 U of FastAP thermosensitive alkaline phosphatase (Thermo Fisher Scientific, EF0651) was added to the reaction and FastAP buffer was added to a 1× final concentration before incubation for 2 h at 37 °C. For DNA, genomic DNA was purified from cells according to the standard protocol of the Monarch Genomic DNA Purification Kit (NEB, T3010S). An additional RNase A (Thermo Fisher Scientific, EN0531) digestion step was performed on the purified DNA and the reaction was recovered with DNA Clean & Concentrator-5 (Zymo Research, D4014). Then, 200 ng DNA was digested with Nucleoside Digestion Mix (NEB, M0649S) at 37 °C for 2 h.

The samples were diluted and filtered (0.22 μm, Millipore) and injected into a C18 reversed-phase column coupled online to the Agilent 6460 LC–MS/MS spectrometer in positive electrospray ionization mode. The nucleosides were quantified using retention time and the nucleoside to base ion mass transitions (for RNA: 268 to 136 for A; 284 to 152 for G; 258 to 126 for m^5^C and 274 to 142 for hm^5^C; for DNA: 228 to 112 for dC, 242 to 126 for 5mdC, 258 to 142 for 5hmdC). Quantification was performed by comparing with the standard curve obtained from pure nucleoside standards running with the same batch of samples.

### Chromatin-associated RNA-seq

Chromatin-associated RNA-seq analyses of mES cells, K-562 and HSPCs were performed similarly. After caRNA isolation, ERCC RNA spike-in mix (Invitrogen, 4456740) was added to purified total caRNA according to the ratio recommended by the standard protocol. Ribosomal RNA was depleted from isolated chromatin-associated RNA with RiboMinus Eukaryote System v2 (Invitrogen, A15026) followed by size-selection using the standard protocol of RNA Clean & Concentrator-5 (RCC-5, Zymo Research, R1013). RNA libraries were constructed with SMARTer Stranded Total RNA-Seq Kit v2 - Pico Input Mammalian (TaKaRa Bio, 634411) according to the manufacturer’s instructions. Three replicates were performed for each condition. Libraries were sequenced on the NovaSeq 6000 sequencer.

### ATAC–seq analysis

ATAC–seq was performed using the ATAC–seq kit (Active Motif, 53150) according to the manufacturer’s instructions. In brief, 50,000 to 100,000 cells were aliquoted for each replicate and mixed with equal amounts of *Drosophila* spike-in (Active Motif, 53154). Cells were then permeabilized with buffer containing 0.1% Tween-20 and 0.01% Digitonin, both supplied by the original kit. Accessible chromatin regions were tagged with pre-assembled Tn5 transposome. Tagged genomic DNA was extracted from cells and DNA libraries were obtained by PCR amplification. Pooled libraries were sequenced on the NovaSeq 6000 sequencer. For ATAC–qPCR, tagged genomic DNA was extracted and amplified by PCR for 8 cycles using the indexing primers from the original kit. Amplified DNAs were subjected to qPCR analysis using individual primer sets.

### m^5^C methylated RNA immunoprecipitation with spike-in

m^5^C modified or unmodified mRNA spike-ins were in vitro transcribed from firefly luciferase or *Renilla* luciferase coding sequences with mMESSAGE mMACHINE T7 Transcription Kit (Invitrogen, AM1344) and manually reconstituted dNTP mixes with 20% m^5^CTP/CTP ratio. 5-methylcytidine-5-triphosphate was obtained from TriLink Biotechnologies (N-101405). Yielded RNA was purified by using the standard protocol of RNA Clean & Concentrator-5 (Zymo Research, R1013). The spike-in RNA mixes were then applied to RNA before fragmentation.

Total RNAs from whole cell or the chromatin-associated fractions were randomly fragmented by incubation at 94 °C for 4 min using 1× fragmentation buffer (NEB, E6186A). Fragmentation was stopped by adding 1× stop solution. Spike-in RNAs were added to each sample. Then, 4 μg anti-m^5^C antibody (Diagenode, MAb-081-100) was conjugated with 30 μl of protein G beads (Invitrogen, 1003D) in 300 μl IP buffer (10 mM Tris-HCl pH 7.5, 150 mM NaCl, 0.05% Triton X-100 (v/v), 1 mM spermidine) for 2 h at 4 °C on a rotating wheel. The same procedure was performed for a control reaction using mouse IgG isotype control (Abcam, ab37355). Bead–antibody complexes were washed three times with IP buffer and finally brought to 250 μl with IP buffer. After heat denaturation and quick chill on ice, 10 μg samples of RNA were added to the bead–antibody complexes and incubated with 1 μl SUPERase•In RNase Inhibitor (Invitrogen, AM2694) overnight at 4 °C on a rotating wheel. After several washes with IP buffer, RNA was incubated in 100 μl elution buffer (5 mM Tris-HCl pH 7.5, 1 mM EDTA, 0.05% SDS, and 200 μg proteinase K (Invitrogen, 25530049)) for 1 h at 50 °C. Beads were removed by centrifugation in a microcentrifuge, and the supernatant was purified with RCC-5 without size selection. Immunoprecipitated RNAs were eluted in water and then analysed using RT–qPCR. For next-generation sequencing, the immunoprecipitated RNAs were used as inputs for library constructions with the SMARTer Stranded Total RNA-Seq Kit v2—Pico Input Mammalian (TaKaRa Bio, 634411) according to the manufacturer’s instructions. Libraries were sequenced on the NovaSeq 6000 sequencer.

For analysing the effects of GC ratio and m^5^C modification levels, we designed three different in vitro transcription templates to get 70%, 50% or 30% GC ratio RNA products based on firefly luciferase mRNA (Supplementary Table 2). DNA oligos were purchased from Integrative DNA Technologies and annealed with a complementary DNA oligo (T7; Supplementary Table 2) to enable T7 DNA polymerase binding. In vitro transcription was performed using the mMESSAGE mMACHINE T7 Transcription Kit (Invitrogen, AM1344) and manually reconstituted dNTP mixes with a 0%, 0.2%, 2% or 20% m^5^CTP/CTP ratio. 5-methylcytidine-5-triphosphate was obtained from TriLink Biotechnologies (N-101405). Yielded RNA was purified using the standard protocol of the RNA Clean & Concentrator-5 (Zymo Research, R1013) kit. meRIP–qPCR experiments were performed according to the protocol mentioned above, and yeast tRNA (Invitrogen, AM7119) was mixed with RNA probes as a carrier.

### RNA amplicon bisulfite sequencing

caRNAs were isolated from *Tet2* WT or *Tet2-*KO mES cells as aforementioned. Ultrafast bisulfite (UBS) conversion was performed according to the published protocol^28^. Reverse transcription was then performed with SuperScript III Reverse Transcriptase (Invitrogen, 18080093) using individual RT primers (Supplementary Table 2). The resulting cDNA was amplified for 10 cycles using NEBNext Ultra II Q5 Master Mix (NEB, M0544S) according to the standard protocol except that the *T*_m_ was set to 50 °C. Amplified DNA was quantified using the universal p5 primer (Supplementary Table 2) and p7 primer from NEBNext Multiplex Oligos for Illumina (NEB, E7500S). cDNAs amplified from different amplicons were then pooled together based on qPCR quantifications to achieve equal sequencing depth in the final DNA library. A final amplification was performed using the two primers (universal p5 primer and p7 primer from NEBNext Multiplex Oligos for Illumina) for 15 cycles using NEBNext Ultra II Q5 Master Mix (NEB, M0544S). PCR products were recovered using 1.0 volume of AMPure XP beads (Beckman Coulter, A63882) and subjected to sequencing on a NovaSEQ-X sequencer.

### meDIP analysis

For methyl-DNA immunoprecipitation (meDIP) analysis, genomic DNA was extracted from cultured cells using the Monarch Genomic DNA Purification Kit (New England Biolabs, T3010S). Unmethylated lambda DNA (Promega, D1521) was spiked at a 0.5% ratio for quality control of the immunoprecipitation. DNAs were then fragmented to 200–1,000 bp by incubation for 22 min with NEBNext dsDNA Fragmentase (New England Biolabs, M0348S). The fragmented DNA was then denatured at 95 °C for 5 min and immediately cooled on ice for another 5 min. The input samples were removed and saved on ice for later use. The reaction was conducted in IP buffer (150 mM NaCl, 10 mM Tris-HCl, pH 7.5, 0.1% NP-40) at 4 °C overnight. The beads were then washed three times with IP buffer, followed by three washes by high-salt wash buffer (500 mM NaCl, 10 mM Tris-HCl, pH 7.5, 0.1% NP-40). Immunoprecipitated DNA was extracted by proteinase K digestion (Invitrogen, 25530049) before qPCR analysis. High-throughput sequencing libraries were constructed using xGen Methyl-Seq Lib Prep kits (IDT, 10009860) and sequenced on the NovaSEQ-X sequencer.

### RNA synthesis rate assay

The RNA synthesis rate was measured with a procedure modified from the protocol Click-iT Nascent RNA Capture Kit, for gene expression analysis (Invitrogen, C10365). mES cells were seeded to 6 cm dishes at the same density in three replicates. After 42 h, cells were treated with 1 mM 5-ethynyl uridine for 10 min, 20 min and 40 min before RNA collection using TRIzol Reagent (Invitrogen, 15596026). Ribosomal RNA was depleted from total RNA preps before the click reaction with biotin azide (PEG4 carboxamide-6-azidohexanyl biotin). Biotinylated RNA was enriched using Dynabeads MyOne Streptavidin T1 (Invitrogen, 65601). ERCC RNA spike-in mix (Invitrogen, 4456740) was added to the eluted RNA with the amount proportional to the total RNA of each sample before rRNA depletion. Spiked RNAs were used as an input for RNA-seq library construction using the SMARTer Stranded Total RNA-Seq Kit v2—Pico Input Mammalian (TaKaRa Bio, 634411) according to the manufacturer’s instructions. Libraries were sequenced on the NovaSeq 6000 sequencer.

### CUT&Tag analysis

Cleavage under targets and tagmentation (CUT&Tag) analysis was performed using the CUT&Tag-IT Assay Kit (Active motif, 53160) according to the manufacturer’s instructions. In brief, 0.2 million cells were used as an input for one replicate and washed with 1× wash buffer. Washed cells were conjugated to concanavalin A beads and permeabilized with Digitonin-containing buffer before incubation with primary antibodies (anti-H3K27me3, anti-H2AK119ub or normal rabbit IgG). Preassembled protein A-Tn5 transposome-enabled DNA tagmentation was performed after secondary antibody conjugation. Equal amounts of *Drosophila* spike-in chromatin preps (Active Motif, 53083) were added to each samples and subjected to the Tn5 tagmentation reaction. Tagged DNA was extracted by proteinase K digestion and amplified by PCR with indexed primers to yield DNA libraries. DNA libraries were subjected to qPCR analysis with gene-specific primers or high-throughput sequencing on the NovaSeq 6000 sequencer.

### Construction of induced tethering mES cell lines

Cell lines stably expressing dCas13 protein fusion with catalytic domain of mouse TET2 (TET2-CD) or catalytic dead mutants were constructed first from WT mES cells. The coding sequence of dCas13 was cloned from plasmid pCMV-dCas13-M3nls, which was a gift from D. Liu (Addgene plasmid, 155366). The coding sequence of TET2-CD was cloned from the plasmid pcDNA3-FLAG-mTET2 (CD), which was a gift from Y. Xiong (Addgene plasmid, 89736), and the catalytic-dead mutant was cloned from the plasmid pcDNA3-Flag-Tet2 CD Mut, which was a gift from Y. Zhang (Addgene plasmid, 72220). pLR5-CBh-dCas9-hEzh2-IRES-Hyg was a gift from H. Ochiai (Addgene plasmid, 122375). The coding sequences of TET2-CD (or mutant) and dCas13 or dCas9 were fused. The fusion protein was delivered to mES cells with the piggyBac transposon system using the pLR5 vector and selected with hygromycin B (Gibco, 10687010). Sequences expressing guide RNA for dCas13 were cloned into a plasmid expressing a Tet operator controlled H1 operator (H1-2O2)^52^. This tet-pLKO-sgRNA-puro plasmid was a gift from N. Gray (Addgene plasmid, 104321). The guide-RNA expression plasmid was delivered into the TET2-CD-fusion protein-expressing mES cells by lentivirus. The resulting cell lines were selected with puromycin (Gibco, A1113803).

### ASO and plasmid transfection in HSPCs

The steric-blocking antisense oligonucleotides (ASOs) (Integrated DNA Technologies) targeted to the hypermethylated motifs were fully modified with 2′-O-methoxyethyl (2′MOE) bases and phosphorothioate bonds, which were also incorporated with a fluorescent dye Cy5 at the 3′ end to monitor transfection efficiency. The NC5 ASO was used as a negative control that was not targeted to the human or mouse genome.

IAPEz-int 2′MOE: AGTTGAATCCTTCTTAACAGTCTGCTTTACGGGAAC

Sequence: /52MOErA/*/i2MOErG/*/i2MOErT/*/i2MOErT/*/i2MOErG/*/i2MOErA/*/i2MOErA/*/i2MOErT/*/i2MOErC/*/i2MOErC/*/i2MOErT/*/i2MOErT/*/i2MOErC/*/i2MOErT/*/i2MOErT/*/i2MOErA/*/i2MOErA/*/i2MOErC/*/i2MOErA/*/i2MOErG/*/i2MOErT/*/i2MOErC/*/i2MOErT/*/i2MOErG/*/i2MOErC/*/i2MOErT/*/i2MOErT/*/i2MOErT/*/i2MOErA/*/i2MOErC/*/i2MOErG/*/i2MOErG/*/i2MOErG/*/i2MOErA/*/i2MOErA/*/i2MOErC//3Cy5Sp/

MERVL 2′MOE: ACCATTACTGGGTATGTTAT

Sequence: /52MOErA/*/i2MOErC/*/i2MOErC/*/i2MOErA/*/i2MOErT/*/i2MOErT/*/i2MOErA/*/i2MOErC/*/i2MOErT/*/i2MOErG/*/i2MOErG/*/i2MOErG/*/i2MOErT/*/i2MOErA/*/i2MOErT/*/i2MOErG/*/i2MOErT/*/i2MOErT/* /i2MOErA/*/i2MOErT//3Cy5Sp/

NC5 2′MOE: GCGACTATACGCGCAATATG

Sequence: /52MOErG/*/i2MOErC/*/i2MOErG/*/i2MOErA/*/i2MOErC/*/i2MOErT/*/i2MOErA/*/i2MOErT/*/i2MOErA/*/i2MOErC/*/i2MOErG/*/i2MOErC/*/i2MOErG/*/i2MOErC/*/i2MOErA/*/i2MOErA/*/i2MOErT/*/i2MOErA/* /i2MOErT/*/i2MOErG//3Cy5Sp/

The crRNA targeting the primary m^5^C sites on IAPEz sequence based on our RNA bisulfite sequencing results was custom-synthesized and cloned into the pLentiRNAGuide_002-hU6-RfxCas13d-DR-BsmBI-EFS-EGFP:P2A:Puro-WPRE vector. The catalytic domain of mouse TET2 (mTET2-CD) or a catalytically dead mutant TET2(H1304Y/D1306A) (mTET2CDHxDCD) was cloned into the pLV[Exp]-[EF-1sc>[NLS-RfxCas13d]:[Linker]:P2A:mCherry(ns):T2A:Bsd vector. All of these plasmids were synthesized, constructed and confirmed by VectorBuilder.

All of the ASOs and plasmids were transfected into HSPCs using electroporation with the P3 Primary Cell 4D-Nucleofector X Kit S (Lonza Bioscience, V4XP-3032) with the program CV-137.

### ASO transfections

We designed ASOs targeting the primary m^5^C sites on IAPEz or MERVL sequences based on our RNA m^5^C sequencing results. ASO transfections in mES cells were performed using the Lipofectamine RNAiMAX Transfection Reagent (Invitrogen, 13778075) according to the manufacturer’s instructions.

### Cross-linking and immunoprecipitation and PAR-CLIP

Cultured mES cells or human leukaemia cells (SKM-1, WT and *TET2*^*−/−*^ THP-1 and K-562) were UV cross-linked at 254 nm with a Stratalinker (Stratagene) twice to achieve a 4,500 J m^−2^ UV flux and then flash-frozen in liquid nitrogen. For photoactivatable ribonucleoside-enhanced crosslinking and immunoprecipitation (PAR-CLIP), 4-thiouridine was added to the cell culture medium 14 h before UVA irradiation (365 nm) three times, 1,500 J m^−2^ each. The pellets were thawed on ice and resuspended in 3 volumes of ice-cold CLIP lysis buffer (50 mM HEPES pH 7.5, 150 mM KCl, 2 mM EDTA, 0.5% (v/v) NP-40, 0.5 mM DTT, 1 × Halt protease and phosphatase inhibitor cocktail (Thermo Fisher Scientific, 78442), 1 × RNaseOUT recombinant ribonuclease inhibitor (Invitrogen, 10777019)). The pellets were lysed by rotating at 4 °C for 15 min after passing through a 26 G needle (BD Biosciences). Embryo suspensions were sonicated on the Bioruptor system (Diagenode) with 30 s on/30 s off for 5 cycles. Lysates were cleared by centrifugation at 21,000*g* for 15 min at 4 °C on a benchtop centrifuge. The supernatants were applied to Flag-antibody-conjugated (Abcam, ab205606) protein A beads (Invitrogen, 1001D) and left overnight at 4 °C on an end-to-end rotor. The beads were washed extensively with 1 ml wash buffer (50 mM HEPES pH 7.5, 300 mM KCl, 0.05% (v/v) NP-40, 1 × Halt protease and phosphatase inhibitor cocktail, 1 × RNaseOUT recombinant ribonuclease inhibitor) at 4 °C five times. Protein–RNA complex conjugated to the beads was treated with 8 U μl^−1^ RNase T1 (Thermo Fisher Scientific, EN0541) at 22 °C for 10 min with shaking. The input samples were digested in parallel. Then, input and IP samples were separated on an SDS–PAGE gel and gel slices at corresponding size ranges were treated by proteinase K (Invitrogen, 25530049) elution. RNA was recovered with TRIZol reagent (Invitrogen, 15596026). T4 PNK (Thermo Fisher Scientific, EK0031) end repair was then performed with purified RNA before library construction with the NEBNext Small RNA Library Prep Set for Illumina (NEB, E7330S). Libraries were pooled and sequenced on the NovaSeq 6000 sequencer.

### Electrophoretic mobility shift assay

Recombinant MBD6-MBD–MBP–His protein was purified from *Escherichia coli* BL21 (DE3). Different concentrations of proteins were mixed with 100 nM FAM-labelled oligo probes in 1 × binding buffer (20 mM HEPES pH 7.5, 40 mM KCl, 10 mM MgCl_2_, 0.1% Triton X-100, 10% glycerol and 1 × RNaseOUT Recombinant Ribonuclease Inhibitor (Invitrogen, 10777019)). The probe–protein mixture was incubated on ice for 30 min. The mixtures were loaded to a 10% Novex TBE Gel (Invitrogen, EC62755BOX). After gel running at 4 °C in 0.5× TBE for 2 h, the gel was washed twice in 0.5× TBE for 5 min. Washed gel was imaged with the GelDoc imaging system (Bio-Rad) with channel ‘FAM’. Individual *K*_D_ values were determined from a regression equation *Y* = [*P*]/(*K*_D_ + [*P*]), where *Y* is the fraction of probe bound at each protein concentration. The fraction bound is determined from the background-subtracted signal intensities using the expression: bound/(bound + unbound). [*P*] is protein concentration in each sample.

### Quantitative analysis of RNA modification levels of CLIP RNA

Cultured mES cells were washed twice with DPBS before UV cross-linking at 254 nm with a Stratalinker (Stratagene) and flash-frozen in liquid nitrogen. The pellets were thawed on ice and resuspended in 3 volumes of ice-cold CLIP lysis buffer (50 mM HEPES pH 7.5, 150 mM KCl, 2 mM EDTA, 0.5% (v/v) NP-40, 0.5 mM DTT, 1 × Halt protease and phosphatase inhibitor cocktail (Thermo Fisher Scientific, 78442), 1 × RNaseOUT recombinant ribonuclease inhibitor (Invitrogen, 10777019)). The pellets were lysed by rotating at 4 °C for 15 min after passing through a 26 G needle (BD Biosciences). The cell suspensions were sonicated on the Bioruptor system (Diagenode) with 30 s on/30 s off for 5 cycles. Lysates were cleared by centrifugation at 21,000*g* for 15 min at 4 °C on a benchtop centrifuge. The supernatants were applied to Flag-antibody (Abcam, ab205606) conjugated protein A beads (Invitrogen, 1001D) and left overnight at 4 °C on an end-to-end rotor. Beads were washed extensively with 1 ml wash buffer (50 mM HEPES pH 7.5, 300 mM KCl, 0.05% (v/v) NP-40, 1 × Halt Protease and Phosphatase Inhibitor Cocktail, 1 × RNaseOUT Recombinant Ribonuclease Inhibitor) at 4 °C five times. Then, the input and IP samples were treated by proteinase K (Invitrogen, 25530049) to release cross-linked RNA. RNA was recovered with TRIZol reagent (Invitrogen, 15596026). Ribosomal RNA was then removed using the RiboMinus Eukaryote System v2 (Invitrogen, A15026) with purification and size-selection using the RNA Clean & Concentrator-5 (Zymo Research, R1013) kit. Recovered RNAs were subjected to digestion and MS/MS analysis.

### Biotinylation of immunoprecipitated RNAs

Biotin labelling of immunoprecipitated RNA was performed according to a published protocol^53^.

### Fluorescence microscopy

For immunolabelling, cells were fixed with 4% PFA in DPBS at 37 °C for 5 min, permeabilized with methanol at −20 °C for 8 min, dried at room temperature for 10 min and then washed three times with DPBS at room temperature. The chambers were blocked in blocking buffer (DPBS, 0.5% BSA, 0.05% Triton X-100, 1:100 SUPERase·In (Invitrogen, AM2694)) for 1 h at room temperature and primary antibodies were diluted in blocking solution according to the suggested fold from the manufacturer’s and incubate at room temperature for 1 h. Chambers were washed three times with 0.05% Triton X-100 in DPBS, then 1:1,000 diluted goat anti rabbit IgG-AF568 conjugate (Invitrogen, A-11011) in blocking solution was added to each well and the chambers were incubated at room temperature for 1 h. The chambers were then washed three times with 0.05% Triton X-100 in DPBS and fixed with 4% PFA in DPBS for 30 min at room temperature and washed three times with DPBS. Nuclei were counterstained with 2 µg ml^−1^ Hoechst 33342 (Abcam, ab145597) in DPBS at room temperature for 20 min, wash with DPBS three times. The chambers were stored at 4 °C before proceeding to imaging on a Leica SP8 laser-scanning confocal microscope at University of Chicago.

### Lifetime profiling

Transcription inhibitor actinomycin D (Act D, Abcam ab141058) was applied to a final concentration of 2.5 μM in mES cell medium to cultured mES cells or cultured Lin^−^KIT^+^ mouse HSPCs. Actinomycin D treatment started at 48 h after siRNA transfection (if any). RNAs were extracted from cells at different timepoints after actinomycin D treatment (10 min, 3 h and 6 h). Custom spike-in RNA (in vitro transcribed from firefly luciferase coding sequence) was added proportional to the yield of total RNA for different samples for RNA quantifications. RNA abundance was normalized to the value at 10 min for each condition.

### DNA-seq data analysis

Raw reads were trimmed with Trimmomatic (v.0.39)^54^ and then mapped to mouse genome (mm10) or human genome (hg38), together with *Drosophila melanogaster* chromatin (spike-in chromatin), using bowtie2 (v.2.4.1)^55^ using the default mode, where multiple alignments are searched and the best one is reported. Mapped reads were deduplicated using the Picard tool MarkDuplicates (v.2.26.2; http://broadinstitute.github.io/picard/).

For ATAC–seq, reads that mapped to the mitochondrial genome were discarded before deduplication. Peaks were identified using MACS2^56^ with the default mode, except for the parameters ‘--shift −75 --extsize 150 --nomodel --call-summits’. For CUT&Tag–seq, peaks were called using MACS2 with the default mode, except for the parameters ‘--broad --broad-cutoff 0.01’. For both ATAC–seq and CUT&Tag-seq, peaks that appeared in at least two biological replicates were retained for subsequent downstream analyses. The chromatin accessibility (ATAC) and H2AK119ub levels (CUT&Tag) were normalized by considering both sequencing depth and spike-in *Drosophila melanogaster* chromatin.

For meDIP–seq, differentially methylated regions were identified using MEDIPS^57^ with the following settings: diff.method = ‘edgeR’, p.adj = ‘bonferroni’, MeDIP = True, CNV = False, minRowSum = 10. Regions with an adjusted *P* value of less than 0.1 were defined as significantly differentially methylated regions.

### Nascent RNA-seq data analysis

Raw reads were trimmed with Trimmomatic (v.0.39)^54^, and then aligned to mouse genome and transcriptome (mm10, version M19) as well as external RNA Control Consortium (ERCC) RNA spike-in control (Thermo Fisher Scientific) using HISAT2 (v.2.2.1)^58^. Annotation files (version M19 for mouse) were obtained from GENCODE database (https://www.gencodegenes.org/)^59^. Reads on each GENCODE annotated gene were counted using HTSeq (v.0.12.4)^60^ and then normalized to counts per million (CPM) using edgeR packages in R^61^. CPM was converted to attomole by linear fitting of the RNA ERCC spike-in. The RNA level and EU adding time were fitted using a linear mathematical model, and the slope was estimated as transcription rate of RNA.

### CLIP–seq data analysis

Low-quality reads were filtered using ‘fastq_quality_filter’, and adapters were clipped using ‘fastx_clipper’, then adapter-free reads were collapsed to remove PCR duplicates using ‘fastx_collapser’ and, finally, reads longer than 15 nucleotides were retained for further analysis (http://hannonlab.cshl.edu/fastx_toolkit/). Reads from rRNA were removed. The preprocessed reads were mapped to mouse genome (mm10) using bowtie (v.1.0.0)^62^ with ‘-v 3 -m 10 -k 1 --best --strata’ parameters. Mapped reads were separated by strands with samtools (v.1.16.1)^63^ and peaks on each strand were called using MACS2 (v.2)^56^ with parameter ‘-nomodel, --keep-dup 5, -g 1.3e8, -extsize 150’ separately. Significant peaks with *q* < 0.01 identified by MACS2 were considered. Peaks identified in at least two biological replicates were merged using bedtools (v.2.31.0)^63^ and were used in the following analyses.

### RNA-seq data analysis

Raw reads were trimmed with Trimmomatic (v.0.39)^54^, then aligned to mouse (mm10) or human (hg38) genome and their corresponding transcriptome, together with external RNA Control Consortium (ERCC) RNA spike-in control (Thermo Fisher Scientific) when applicable, using HISAT2 (v.2.2.1)^58^. Annotation files (version M19 for mouse, and version v29 for human in gtf format) were obtained from GENCODE database (https://www.gencodegenes.org/)^59^. Reads were counted for each GENCODE annotated gene using HTSeq (v.0.12.4)^60^ and for caRNAs using featureCounts^64^, and then differentially expressed genes were called using DESeq2 package in R^65^ with *P* < 0.05. In this step, the spike-in normalization factor was calculated by dividing the number of reads mapped to ERCC spike-ins by the total number of mapped transcriptomic reads. This factor was then included in the size factor calculation for DESeq2.

### m^5^C meRIP–seq data analysis

Raw reads were trimmed with Trimmomatic (v.0.39)^54^, then aligned to mouse (mm10) or human (hg38) genome and transcriptome, together with m^5^C modified or unmodified mRNA spike-ins (see the ‘m^5^C methylated RNA immunoprecipitation with spike-in’ section for details), using HISAT2 (v.2.1.0)^58^. Annotation files (version M19 for mouse, and version v29 for human in gtf format) were downloaded from the GENCODE database (https://www.gencodegenes.org/)^59^. Mapped reads were deduplicated using a Picard tool ‘MarkDuplicates’ (v.2.26.2) (http://broadinstitute.github.io/picard/). The remaining reads were separated by strands with samtools (v.1.16.1)^63^ and peaks on each strand were called using MACS2 (v.2)^56^ with the parameters ‘--nomodel --extsize 150’. Genome-specific parameters ‘-g hs’ for human and ‘-g mm’ for mouse were separately applied. We required significant peaks (*q* < 0.01) to appear in all biological samples to be considered validated. Peaks within the same conditions were then merged using bedtools (v.2.31.0)^63^ for subsequent analysis. To quantify m^5^C methylation levels, we initially compared reads mapped to m^5^C-methylated spike-ins with those mapped to their unmethylated counterparts to confirm satisfactory pull-down efficiency. The m^5^C methylation levels were determined by calculating the log_2_-transformed fold changes between immunoprecipitation) and input samples. The normalization factor was calculated by dividing the number of reads mapped to the m^5^C-methylated spike-in by the total number of transcriptomic reads. This approach enabled us to quantify the global changes in m^5^C levels under different conditions.

### Chromatin-associated RNA UBS amplicon-seq analysis

Adapter sequences and low-quality reads were trimmed using cutadapt (v.4.0). Only properly paired reads with a length less than 20 nucleotides were retained. The 7 nucleotides of the UMI at the 5′ end of the insert fragments (R2) were extracted. Clean reads were then mapped to the mouse genome sequence (mm10) using the HISAT-3n tool^66^ with the ‘--base-change C,T’ argument. To leverage the strand-specific property of the library, the ‘--directional-mapping’ parameter was applied. To increase the accuracy of site identification, only properly paired reads without soft clipping were retained. To eliminate unconverted clusters, reads containing more than three unconverted C sites, or where more than one-third of the total C sites were unconverted, were discarded. A binomial model was used to calculate a *P* value for each site, and sites with a *P* value less than 0.01 were classified as m^5^C sites.

### Antibodies

The antibodies used in this study are summarized below: rabbit monoclonal anti-H2AK119ub antibody (Cell Signaling Technology, 8240S, 1:1,000 for western blot, 1:50 for CUT&Tag); rabbit monoclonal anti-H3 antibody (Cell Signaling Technology, 4499S, 1:1,000); mouse monoclonal anti-TET2 antibody (MilliporeSigma, MABE462, 1:500); rabbit monoclonal anti-GAPDH antibody, HRP conjugate (Cell Signaling Technology, 8884S, 1:1,000); rabbit monoclonal anti-DDDDK tag antibody (Abcam, ab205606, 1:1,000 for western blot, 1:50 for immunoprecipitation); rabbit polyclonal anti-SNRP70/U1-70K antibody (Abcam, ab83306, 1:1,000); mouse monoclonal anti-5-methylcytosine antibody (Diagenode, C15200081-100, 1:1,000 for dot blot, 1:50 for meRIP); mouse monoclonal anti-hm^5^C antibody (Diagenode, C15200200-100, clone Mab-31HMC, 1:1,000 for dot blot); rabbit monoclonal anti-H3K27me3 antibody (Cell Signaling Technology, 9733S, only for CUT&Tag experiments, 1:50); mouse monoclonal anti-BAP1 antibody (Santa Cruz, sc-28383, 1:50 for CUT&Tag). Goat anti-rabbit IgG, HRP conjugated antibody (Cell Signaling Technology, 7074S, 1:2,000) and horse anti-mouse IgG, HRP conjugated antibody (Cell Signaling Technology, 7076S, 1:2,000) were used as secondary antibodies. Mouse IgG-isotype control (Abcam, ab37355, 1:50 for immunoprecipitation) and rabbit IgG-isotype control (Abcam, ab37415, 1:50 for immunoprecipitation) were used as normal IgG controls. PerCP-Cy5.5 mouse lineage antibody cocktail (BD Biosciences, 561317, 1:100); PE rat anti-mouse CD117 (BD Biosciences, 553869, 1:100); Brilliant Violet 421 (BV421, 1:100) anti-mouse/human CD11b (Mac-1) (BioLegend, 101236, 1:100); APC mouse anti-human CD45 (BD Biosciences, 555485, 1:100); PE mouse anti-human CD33 (BD Biosciences, 561816, 1:100); PE-Cy7 rat anti-mouse CD45 (BD Biosciences, 552848, 1:100); PerCP-Cy5.5 mouse anti-mouse CD45.2 (BD Biosciences, 552950, 1:100) and FITC mouse anti-mouse CD45.1 (BD Biosciences, 553775, 1:100). All antibodies were applied at a dilution fold according to the manufacturer’s suggestions for specific use unless specified elsewhere in the Methods.

### Reporting summary

Further information on research design is available in the Nature Portfolio Reporting Summary linked to this article.

## Online content

Any methods, additional references, Nature Portfolio reporting summaries, source data, extended data, supplementary information, acknowledgements, peer review information; details of author contributions and competing interests; and statements of data and code availability are available at 10.1038/s41586-024-07969-x.

## Supplementary information

Supplementary Information Supplementary Figs. 1–3.

Reporting Summary

Supplementary Table 1 Summary of high-throughput sequencing samples. Antibodies and library sources were indicated.

Supplementary Table 2 Sequences of qPCR primers, siRNAs, oligo nucleotides, antisense oligos and guide RNAs. Experiment type and references were indicated.

## Source data

Source Data Figs. 1–5 and Source Data Extended Data Figs. 1–9

## Data Availability

High-throughput sequencing data can be accessed at the GEO under accession number GSE241347. Previously published sequencing data that were reanalysed here are available under accession codes GSE103269, GSE48518, GSE181698 and GSE48519. All other data supporting the findings of this study are available from the corresponding author on reasonable request. Source data are provided with this paper.
