# Supplementary material for: RNA m5C oxidation by TET2 regulates chromatin state and leukaemogenesis
Source: Nature. 2024 Oct 2;634(8035):986–94. doi: 10.1038/s41586-024-07969-x (PMC11499264; doi:10.1038/s41586-024-07969-x)
Supplement: Supplementary file 1 — Supplementary Figs. 1–3. [file 41586_2024_7969_MOESM1_ESM.pdf]

nature portfolio

https://doi.org/10.1038/s41586-024-07969-x

Supplementary information

RNA m^5C oxidation by TET2 regulates chromatin state and leukaemogenesis

In the format provided by the authors and unedited

RNA m^5C oxidation by TET2 regulates chromatin state and leukemogenesis

Zhongyu Zou[1,2,7], Xiaoyang Dou[1,2,7], Ying Li[3,7], Zijie Zhang[1,2], Juan Wang[3], Boyang Gao[2,4], Yu Xiao[1,2], Yiding Wang[2,4], Lijie Zhao[1,2], Chenxi Sun[1,2], Qinzhe Liu[1,2], Xianbin Yu[1,2], Hao Wang[1,2], Juyeong Hong[3], Qing Dai[1,2], Feng-Chun Yang[5,6], Mingjiang Xu[3,6*], and Chuan He[1,2*]

SUPPLEMENTARY INFORMATION

Contents:

Supplementary Figure 1: Uncropped scans with size marker indications

Supplementary Figure 2: Gating strategies for single-colour flow cytometry

Supplementary Figure 3: Gating strategies for multiple-colour flow cytometry

Supplementary Table 1: Summary of high-throughput sequencing samples. Antibodies and library sources were indicated.

Supplementary Table 2: Sequences of qPCR primers, siRNAs, oligo nucleotides, antisense oligos, and guide RNAs. Experiment type and references were indicated.

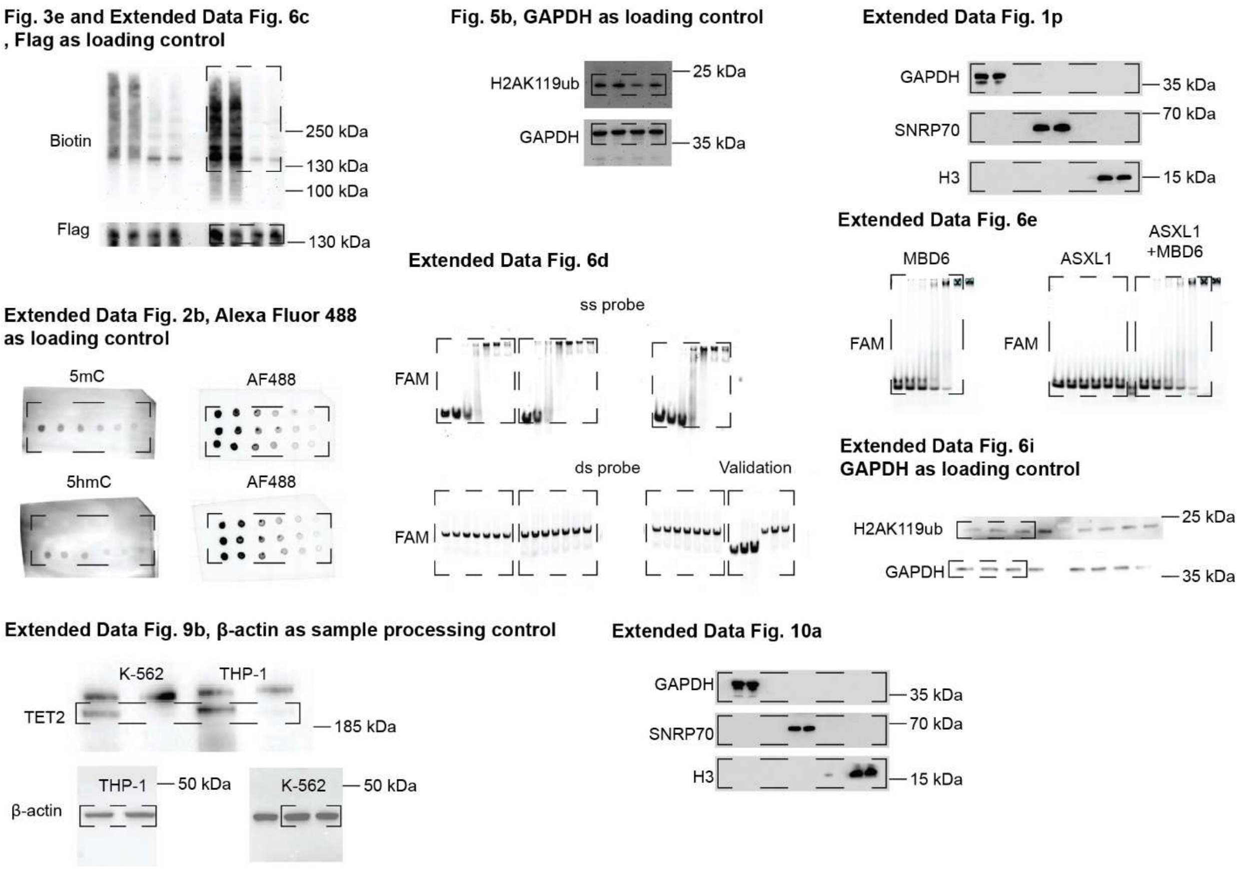

Supplementary Figure 1 | Uncropped scans with size marker indications

Full scans of western blots and EMSA for the indicated figure panels. Dashed boxes indicate areas being cropped and displayed.

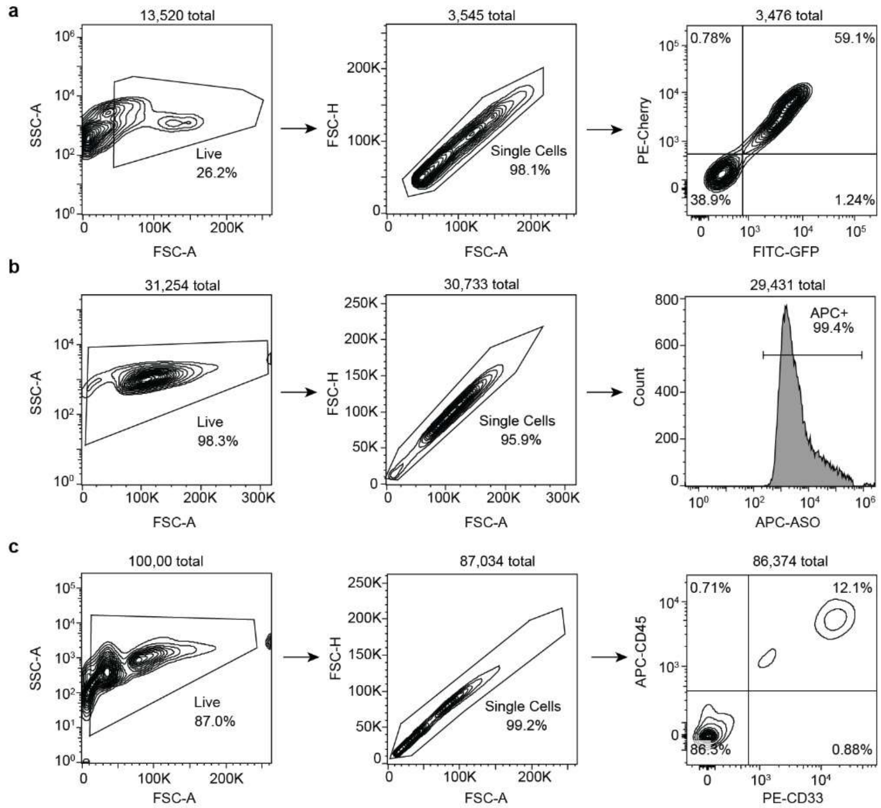

Supplementary Figure 2 | Gating strategies for single-colour flow cytometry

a, Forward scatter (FSC-A) versus side scatter (SSC-A) was set to gate all live hematopoietic cells, but exclude small debris. All single cells are gated by FSC-H/FSC-A, but exclude cell clumps. PE and FITC are used to gate cherry and GFP labeling respectively. For Extended Data Fig. 8b.

b, Forward scatter (FSC-A) versus side scatter (SSC-A) was set to gate all live hematopoietic cells, but exclude small debris. All single cells are gated by FSC-H/FSC-A, but exclude cell clumps. APC is used to gate Cy5 labeled ASO. For Extended Data Fig. 8f.

c, Forward scatter (FSC-A) versus side scatter (SSC-A) was set to gate all live hematopoietic cells, but exclude small debris. All single cells are gated by FSC-H/FSC-A, but exclude cell clumps. Human leukemia donor cells are enriched by human CD45 or CD33. For Extended Data Fig. 9i.

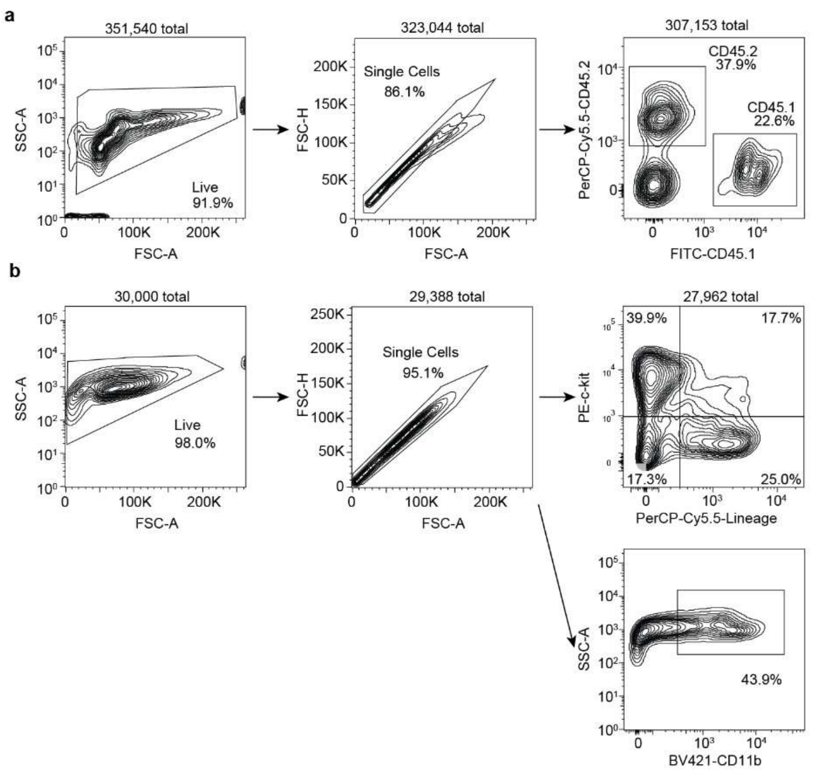

Supplementary Figure 3 | Gating strategies for multiple-colour flow cytometry

a, Forward scatter (FSC-A) versus side scatter (SSC-A) was set to gate all live hematopoietic cells, but exclude small debris. All single cells are gated by FSC-H/FSC-A, but exclude cell clumps. Donor cells are enriched by mouse CD45.2. For Fig. 4b,c.

b, Forward scatter (FSC-A) versus side scatter (SSC-A) was set to gate all live hematopoietic cells, but exclude small debris. All single cells are gated by FSC-H/FSC-A, but exclude cell clumps. Mouse HSPCs cells are enriched by $Lin^{-}c\text{-}kit^{+}$ population. Myeloid cells are enriched by CD11b population. For Fig. 4g, Extended Data Fig. 7d,k, Extended Data Fig. 8d,e, h, i.
