# Supplementary material for: RNA m5C oxidation by TET2 regulates chromatin state and leukaemogenesis
Source: Nature. 2024 Oct 2;634(8035):986–94. doi: 10.1038/s41586-024-07969-x (PMC11499264; doi:10.1038/s41586-024-07969-x)
Supplement: Supplementary file 2 — Reporting Summary [file 41586_2024_7969_MOESM2_ESM.pdf]

nature portfolio

Corresponding author(s): Chuan He and Mingjiang Xu

Last updated by author(s): July,24 2024

Reporting Summary

Nature Portfolio wishes to improve the reproducibility of the work that we publish. This form provides structure for consistency and transparency in reporting. For further information on Nature Portfolio policies, see our Editorial Policies and the Editorial Policy Checklist.

Statistics

For all statistical analyses, confirm that the following items are present in the figure legend, table legend, main text, or Methods section.

n/a	Confirmed	
☐	☒	The exact sample size (*n*) for each experimental group/condition, given as a discrete number and unit of measurement
☐	☒	A statement on whether measurements were taken from distinct samples or whether the same sample was measured repeatedly
☐	☒	The statistical test(s) used AND whether they are one- or two-sided<br>*Only common tests should be described solely by name; describe more complex techniques in the Methods section.*
☒	☐	A description of all covariates tested
☐	☒	A description of any assumptions or corrections, such as tests of normality and adjustment for multiple comparisons
☐	☒	A full description of the statistical parameters including central tendency (e.g. means) or other basic estimates (e.g. regression coefficient) AND variation (e.g. standard deviation) or associated estimates of uncertainty (e.g. confidence intervals)
☐	☒	For null hypothesis testing, the test statistic (e.g. *F*, *t*, *r*) with confidence intervals, effect sizes, degrees of freedom and *P* value noted<br>*Give P values as exact values whenever suitable.*
☒	☐	For Bayesian analysis, information on the choice of priors and Markov chain Monte Carlo settings
☐	☒	For hierarchical and complex designs, identification of the appropriate level for tests and full reporting of outcomes
☐	☒	Estimates of effect sizes (e.g. Cohen's *d*, Pearson's *r*), indicating how they were calculated

Our web collection on statistics for biologists contains articles on many of the points above.

Software and code

Policy information about availability of computer code

Data collection	No software was used for data collection.

Data analysis	Trimmomatic (version 0.39)<br>Bowtie2 (version 2.4.1)<br>Picard tool (version 2.26.2)<br>HISAT2 (version 2.2.1)<br>HTSeq (version 0.12.4)<br>edgeR (version 3.28.1)<br>bowtie (version 1.0.0)<br>fastx_toolkit (http://hannonlab.cshl.edu/fastx_toolkit/, version 0.0.14)<br>MACS2 (version 2)<br>bedtools (version 2.31.0)<br>DESeq2 (version 1.26.0)<br>samtools (version 1.16.1)<br>MEDIPS (version 1.56.0)<br>MarkDuplicates (version 2.26.2)<br>HISAT-3n (version 2.2.1-3n-0.03)<br>featureCounts (Rsubread, version 2.0.1)<br>Cutadapt (version 4.0)

For manuscripts utilizing custom algorithms or software that are central to the research but not yet described in published literature, software must be made available to editors and reviewers. We strongly encourage code deposition in a community repository (e.g. GitHub). See the Nature Portfolio guidelines for submitting code & software for further information.

Data

Policy information about availability of data

All manuscripts must include a data availability statement. This statement should provide the following information, where applicable:

- Accession codes, unique identifiers, or web links for publicly available datasets
- A description of any restrictions on data availability
- For clinical datasets or third party data, please ensure that the statement adheres to our policy

High-throughput sequencing data can be accessed in the Gene Expression Omnibus (GEO) under accession number GSE241347. Previously published sequencing data that were re-analyzed here are available under accession code GSE103269, GSE48518, GSE181698, GSE48519. Source data are provided with this study. All other data supporting the findings of this study are available from the corresponding author on reasonable request.

Research involving human participants, their data, or biological material

Policy information about studies with human participants or human data. See also policy information about sex, gender (identity/presentation), and sexual orientation and race, ethnicity and racism.

Reporting on sex and gender	N/A
Reporting on race, ethnicity, or other socially relevant groupings	N/A
Population characteristics	N/A
Recruitment	N/A
Ethics oversight	N/a

Note that full information on the approval of the study protocol must also be provided in the manuscript.

Field-specific reporting

Please select the one below that is the best fit for your research. If you are not sure, read the appropriate sections before making your selection.

☒ Life sciences ☐ Behavioural & social sciences ☐ Ecological, evolutionary & environmental sciences

For a reference copy of the document with all sections, see nature.com/documents/nr-reporting-summary-flat.pdf

Life sciences study design

All studies must disclose on these points even when the disclosure is negative.

Sample size	No statistical methods were used to predetermine sample sizes. The sample size was determined based on previous studies and literature in the field using similar experimental paradigms (Liu J et al. Science, 2020; Wei J et al. Science, 2022; Dou X et al. Nature Cell Biology, 2023).
Data exclusions	No data were excluded from the analyses.

Replication	All attempts at replication were successful and we did three replicates for most experiments. Number of replicates were stated throughout the manuscript.
Randomization	Samples were allocated into different groups randomly.
Blinding	The investigators were blinded to group allocation during data collection. Data was post hoc registered to the treatment conditions and analyzed to prevent any bias.

Reporting for specific materials, systems and methods

We require information from authors about some types of materials, experimental systems and methods used in many studies. Here, indicate whether each material, system or method listed is relevant to your study. If you are not sure if a list item applies to your research, read the appropriate section before selecting a response.

Materials & experimental systems

n/a	Involved in the study
☐	☒ Antibodies
☐	☒ Eukaryotic cell lines
☒	☐ Palaeontology and archaeology
☐	☒ Animals and other organisms
☒	☐ Clinical data
☒	☐ Dual use research of concern
☒	☐ Plants

Methods

n/a	Involved in the study
☒	☐ ChIP-seq
☐	☒ Flow cytometry
☒	☐ MRI-based neuroimaging

Antibodies

Antibodies used	The antibodies used in this study are summarized below: rabbit monoclonal anti-H2AK119ub antibody (Cell Signaling Technology, 8240S, RRID:AB_10891618, clone D27C4); rabbit monoclonal anti-H3 antibody (Cell Signaling Technology, 4499S, RRID:AB_10544537, clone D1H2); mouse monoclonal anti-TET2 antibody (MilliporeSigma, MABE462, RRID:AB_2923169, clone hT2H); rabbit monoclonal anti-GAPDH antibody, HRP conjugate (Cell Signaling Technology, 8884S, RRID:AB_11129865, clone D16H11); rabbit monoclonal anti-DDDDK tag antibody (Abcam, ab205606, RRID:AB_2916341, clone EPR20018-251); rabbit polyclonal anti-SNRP70/U1-70K antibody (Abcam, ab83306, RRID:AB_10673827); mouse monoclonal anti-5-methylcytosine antibody (Diagenode, C15200081-100, RRID:AB_2572207, clone 33D3); mouse monoclonal anti-5-hydroxymethylcytosine antibody (Diagenode, C15200200-100, clone Mab-31HMC); rabbit monoclonal anti-H3K27me3 antibody (Cell Signaling Technology, 9733S, RRID:AB_2616029, clone C36B11, only for CUT&Tag experiments); mouse monoclonal anti-BAP1 antibody (Santa Cruz, sc-28383, RRID:AB_626723, clone C-4). Goat anti-rabbit IgG, HRP conjugated antibody (Cell Signaling Technology, 7074S, RRID:AB_2099233) and horse anti-mouse IgG, HRP conjugated antibody (Cell signaling Technology, 7076S, RRID:AB_330924) were used as secondary antibodies. Mouse IgG-Isotype Control (Abcam, ab37355, RRID:AB_2665484) and rabbit IgG-Isotype Control (Abcam, ab37415, RRID:AB_2631996) were used as normal IgG controls. PerCP-Cy™5.5 mouse lineage antibody cocktail (BD Biosciences, 561317, RRID:AB_10612020); PE Rat anti-mouse CD117 (BD Biosciences, 553869, RRID:AB_395103); Brilliant Violet 421™ (BV421) anti-mouse/human CD11b (Mac-1) (BioLegend, 101236, RRID:AB_11203704); APC Mouse Anti-Human CD45 (BD Biosciences, 555485, RRID:AB_398600); PE Mouse anti-human CD33 (BD Biosciences, 561816, RRID:AB_10896480, 1:100); PE-Cy™7 Rat anti-mouse CD45 (BD Biosciences, 552848, RRID:AB_394489, 1:100); PerCP-Cy™5.5 Mouse Anti-Mouse CD45.2 (BD Biosciences, 552950, RRID:AB_394528, clone 104(RUO)) and FITC Mouse Anti-Mouse CD45.1 (BD Biosciences, 553775, RRID: AB_395043, clone A20(RUO)). All antibodies were applied at a dilution fold according to the manufacturer's suggestions for specific use unless specified in the methods section.
Validation	All the antibodies were used according to the manufacturer's instructions for the validated purposes, see the links below. RRIDs for all the antibodies used were provided.<br>H2AK119ub: for mouse and human CUT&Tag and WB (https://www.cellsignal.com/products/primary-antibodies/ubiquityl-histone-h2a-lys119-d27c4-xp-rabbit-mab/8240).<br>H3: for mouse and human WB (https://www.cellsignal.com/products/primary-antibodies/histone-h3-d1h2-xp-rabbit-mab/4499).<br>TET2: for mouse WB (https://www.sigmaaldrich.com/US/en/product/mm/mabe462).<br>GAPDH-HRP: for human and mouse WB (https://www.cellsignal.com/products/antibody-conjugates/gapdh-d16h11-xp-174-rabbit-mab-hrp-conjugate/8884).<br>DDDDK tag (Flag tag): for WB and IP (https://www.abcam.com/en-us/products/primary-antibodies/ddddk-tag-binds-to-flag-tag-sequence-antibody-epr20018-251-ab205606).<br>SNRP70: for human and mouse WB (https://www.abcam.com/en-us/products/primary-antibodies/snrp70-u1-70k-antibody-ab83306).<br>5-methylcytosine (5mC): for IP and dot blot (https://www.diagenode.com/en/p/5-mc-monoclonal-antibody-33d3-premium-100-ug-50-ul).<br>5-hydroxymethylcytosine (5hmC): for dot blot (https://www.diagenode.com/en/p/5-hmc-monoclonal-antibody-mouse-classic-50-ug-50-ul).<br>H3K27me3: for WB and CUT&Tag (https://www.cellsignal.com/products/primary-antibodies/tri-methyl-histone-h3-lys27-c36b11-rabbit-mab/9733).<br>BAP1: for IP (https://www.scbt.com/p/bap1-antibody-c-4).<br>Goat anti-rabbit IgG, HRP conjugated antibody: for WB (https://www.cellsignal.com/products/secondary-antibodies/anti-rabbit-igg-hrp-linked-antibody/7074).<br>Horse anti-mouse IgG, HRP conjugated antibody: for WB (https://www.cellsignal.com/products/secondary-antibodies/anti-mouse-

igg-hrp-linked-antibody/7076).
PerCP-Cy™5.5 mouse lineage antibody: for flow (https://www.bdbiosciences.com/en-us/products/reagents/flow-cytometry-reagents/research-reagents/panels-multicolor-cocktails-ruo/percp-cy-5-5-mouse-lineage-antibody-cocktail-with-isotype-control.561317).
PE Rat anti-mouse CD117: for flow (https://www.bdbiosciences.com/en-us/products/reagents/flow-cytometry-reagents/research-reagents/single-color-antibodies-ruo/pe-rat-anti-mouse-cd117.553869).
Brilliant Violet 421™ (BV421) anti-mouse/human CD11b (Mac-1): for flow (https://www.biolegend.com/de-de/products/brilliant-violet-421-anti-mouse-human-cd11b-antibody-7163?GroupID=BLG10427)
APC Mouse Anti-Human CD45: for flow (https://www.bdbiosciences.com/en-us/products/reagents/flow-cytometry-reagents/research-reagents/single-color-antibodies-ruo/apc-mouse-anti-human-cd45.555485).
PE Mouse anti-human CD33: for flow (https://www.bdbiosciences.com/en-us/products/reagents/flow-cytometry-reagents/research-reagents/single-color-antibodies-ruo/pe-mouse-anti-human-cd33.561816).
PE-Cy™7 Rat anti-mouse CD45: for flow (https://www.bdbiosciences.com/en-us/products/reagents/flow-cytometry-reagents/research-reagents/single-color-antibodies-ruo/pe-cy-7-rat-anti-mouse-cd45.552848).
PerCP-Cy™5.5 Mouse Anti-Mouse CD45.2: for flow (https://www.bdbiosciences.com/en-us/products/reagents/flow-cytometry-reagents/research-reagents/single-color-antibodies-ruo/percp-cy-5-5-mouse-anti-mouse-cd45-2.552950).
FITC Mouse Anti-Mouse CD45.1: for flow (https://www.bdbiosciences.com/en-eu/products/reagents/flow-cytometry-reagents/research-reagents/single-color-antibodies-ruo/fitc-mouse-anti-mouse-cd45-1.553775).

Eukaryotic cell lines

Policy information about cell lines and Sex and Gender in Research

Cell line source(s)

WT and Tet2-/- mouse embryonic stem cells (mESCs) were gifts from Dr. Bing Ren lab15,61. The control and KO ESCs have been shown to be pluripotent by chimera formation assay. All mESCs were kept in DMEM (Gibco, 11995065) supplemented with 15% Stem Cell Qualified Fetal Bovine Serum, Heat Inactivated (Gemini Bio Products, 100-525), 1 × L-glutamine (Gibco, 25030081), NEAA (Gibco, 25030081), LIF (MilliporeSigma, ESG1107), 1 × β-mercaptoethanol (Gibco, 21985023), 3 μM CHIR99021 (STEMCELL Technologies, 72052) and 1 μM PD0325901 (STEMCELL Technologies, 72182) at 37 °C and 5% CO2.
For stable TET2 overexpression mESCs, empty vector, WT Tet2 or Tet2 HxD mutant bearing piggyBac plasmids were constructed and transfected into Tet2 KO or Pspc1 KO mESCs using Lipofectamine™ 3000 Transfection Reagent (Invitrogen, L3000001) following the standard protocol. Stable expression clone selection was performed using 0.1 mg/ml hygromycin B (Gibco, 10687-010) for two weeks. The medium was replaced every 24 hours. ES cells were passaged on gelatin-coated plates twice to clear feeder cells before experiments.
Wild-type THP-1, K-562, and TF-1 cells were obtained from the American Type Culture Collection (ATCC). SKM-1 cell line was obtained from DSMZ (German Collection of Microorganisms and Cell Cultures GmbH). Wild-type OCI-AML3 cell was a gift from Dr. Lucy Godley at University of Chicago. Wild-type and TET2-/- K-562 and THP-1 cells were gifts from Dr. Babal K. Jha at Cleveland Clinic as previously generated62. THP-1, K-562, SKM-1, and OCI-AML3 cells were kept in RPMI-1640 (Gibco, 61870036) with 10% fetal bovine serum (FBS, Gibco 26140079) at 37 °C and 5% CO2. TF-1 was kept in RPMI-1640 (Gibco, 61870036) with 10% FBS (Gibco 26140079) and 2 ng/ml recombinant GM-CSF (Peprotech, 300-03) at 37 °C and 5% CO2.
U-87 MG (HTB-14™), LN-229 (CRL-2611™), Hep G2 (HB-8065™), HeLa (CCL-2™), HCT 116 (CCL-247™), A549 (CCL-185™) and A-375 (CRL-1619™) cells were obtained from American Type Culture Collection (ATCC). U-87 MG and LN-229 were kept in ATCC-formulated Eagle's Minimum Essential Medium (ATCC, 30-2003) supplemented with 10% FBS (Gibco, 26140079) and 5% FBS (Gibco, 26140079), respectively. Hep G2, HeLa, HCT 116, A549 and A-375 cells were kept in DMEM (Gibco, 11995065) supplemented with 10% FBS (Gibco 26140079). All cell types were kept at 37 °C and 5% CO2.
shNC and shMBD6 THP-1 and K-562 cell lines were constructed by lentivirus transduction with TransDux™ MAX Lentivirus Transduction Reagent (System Biosciences, LV860A-1). Lentiviral particles were prepared by using HEK293T cells and lentiviral packaging plasmids pCMV-VSV-G and pCMV-dR8.2 (pCMV-VSV-G and pCMV-dR8.2 were gifts from Bob Weinberg (Addgene plasmid # 8454 ; http://n2t.net/addgene:8454 ; RRID:Addgene_8454 and Addgene plasmid # 8455 ; http://n2t.net/addgene:8455 ; RRID:Addgene_8455) and short hairpin RNA (shRNA) plasmid pLKO.1-shC002 (MilliporeSigma, SHC002) or pLKO.1-shMBD6 (MilliporeSigma, TRCN000038787). Forty-eight hours after transfection, lentiviral particles were precipitated with PEG-it Virus Precipitation Solution (System Biosciences, LV810-1). shNC and shMBD6 THP-1 and K-562 cell lines were kept in RPMI-1640 (Gibco, 61870036) with 10% fetal bovine serum (FBS, Gibco) and 1 μg/mL puromycin (Gibco, A1113803) at 37 °C and 5% CO2. siRNA or gene overexpression plasmids transfection in K-562 and THP-1 cells were performed according to the manufacturer's instructions for SF Cell Line 4D-Nucleofector™ X Kit (Lonza Biosciences, V4XC-2032, FF-120 for K-562) or SG Cell Line 4D-Nucleofector™ X Kit (Lonza Biosciences, V4XC-3024, FF-100 for THP-1)
TET2 KO THP-1 cell line for PDX model was generated using CRISPR-Cas9 system. Single-guide RNAs were designed with CRISPick tool (https://portals.broadinstitute.org/gppx/crispick/public) and then cloned into LentiCRISPR V2-GFP vector by Synbio Technologies. THP-1 cells were infected by Lentiviral particles for 72 hours and followed by GFP positive cells selection using BD FACSMelody™ Cell Sorter (BD Biosciences). KO efficiency was verified by Western Blot.
shNC, shMBD6 (MilliporeSigma, TRCN0000178563), shNsun2 (MilliporeSigma, TRCN0000325347), shNsun5 (MilliporeSigma, TRCN0000097512) or shTrdmt1 (MilliporeSigma, TRCN0000328293) LK HSPCs were constructed by electroporation with P3 Primary Cell 4D-Nucleofector™ X Kit S (Lonza Bioscience, Cat#V4XP-3032) by program CV-137.

Authentication

All the cell lines used were directly purchased from the indicated sources and we did not use any commonly misidentified lines. No further authentications were performed.

Mycoplasma contamination

All the cell lines used were tested negative of mycoplasma contamination.

Commonly misidentified lines
(See ICLAC register)

No commonly misidentified cell lines were used in this study.

Animals and other research organisms

Policy information about studies involving animals; ARRIVE guidelines recommended for reporting animal research, and Sex and Gender in Research

Laboratory animals	These mice used in this study were backcrossed for more than 6 generations with C57BL/6 mice. 6–8 weeks old of WT C57BL/6 and Tet2-/- mice were applied throughout this study, including both male and female. The adult NOD.Cg-PrkdcscidIl2rgtm1Wjl/SzJ (NSG) mice (6-8 weeks old) for in vivo xenotransplantation study and 8-week-old BoyJ (CD45.1+) mice for competitive repopulation assay are purchased from The Jackson Laboratory.
Wild animals	No wild animals were used in this study.
Reporting on sex	Similar number of male and female mice were used in the human leukemia cells xenograft studies. Based on our knowledge, sex and leukemogenesis/HSPC maintenance of human leukemia cells in NSG mice are not relevant. We didn't observe significant difference in leukemogenesis/HSPC maintenance and survival between male and female mice.
Field-collected samples	No field collected samples were used in this study.
Ethics oversight	All animal studies were performed with the approval from the Institutional Animal Care and Use Committee (IACUC) at The University of Texas Health Science Center at San Antonio (UTHSCSA) and conducted in accordance with the institutional and national guidelines and regulations.<br>Mouse housing conditions: a 14-hour light/10-hour dark cycle is used. Researchers do not enter the mouse room during the dark cycle. Room temperatures were set to 21°C with 40–60% humidity. Immunodeficient mice (NSG) were housed in the same room condition but in a separate sterile room established for immunodeficient mice at UTHSCSA.

Note that full information on the approval of the study protocol must also be provided in the manuscript.

Flow Cytometry

Plots

Confirm that:

☒ The axis labels state the marker and fluorochrome used (e.g. CD4-FITC).

☒ The axis scales are clearly visible. Include numbers along axes only for bottom left plot of group (a 'group' is an analysis of identical markers).

☒ All plots are contour plots with outliers or pseudocolor plots.

☒ A numerical value for number of cells or percentage (with statistics) is provided.

Methodology

Sample preparation	Cells were stained with PerCP-Cy™5.5 mouse lineage antibody cocktail (BD Biosciences, 561317) and PE Rat anti-mouse CD117 (BD Biosciences, 553869) antibodies for hematopoietic stem and progenitor cells analysis. Brilliant Violet 421™ (BV421) anti-mouse/human CD11b (Mac-1) (BioLegend, 101236) was used to analyze myeloid lineage. Human chimerism was analyzed with PE Mouse anti-human CD33 (BD Biosciences,561816) or APC Mouse Anti-Human CD45 (BD Biosciences, 555485) in peripheral blood (PB) and bone marrow (BM) cells from NSG mice xenotransplanted with K-562 or THP-1 cells. PerCP-Cy™5.5 Mouse Anti-Mouse CD45.2 (BD Biosciences, 552950) and FITC Mouse Anti-Mouse CD45.1 (BD Biosciences, 553775) antibodies were used for analyzing CD45.2/CD45.1 chimeras in competitive repopulation assay.
Instrument	All the analyses were performed with a BD FACSCelesta™ flow cytometer (BD Biosciences)
Software	All flow cytometry data were analyzed by FlowJo-V10 software (TreeStar)
Cell population abundance	For hematopoietic stem and progenitor Linc-Kit+ (LK) cell selection, magnetic-activated cell sorting was applied with autoMACS® Pro Separator (Miltenyi Biotec). Briefly, the lineage-positive cells (Lin+) were depleted from total BM cells of 6–8 weeks old mice using the Direct Lineage Cell Depletion Kit (Miltenyi Biotec, 130-110-470), and then the lineage-negative cells (Lin-) were sorted with c-Kit (CD117) MicroBeads (Miltenyi Biotec, 130-091-224). The purity of selected cells was analyzed by flow cytometry to be > 78%.
Gating strategy	Forward scatter (FSC-A) versus side scatter (SSC-A) was set to gate all live hematopoietic cells, but exclude small debris. All single cells are gated by FSC-H/FSC-A, but exclude cell clumps. The unstained sample and single antibody-stained controls are used to gate where boundaries between “positive” and “negative” staining cell population.

☒ Tick this box to confirm that a figure exemplifying the gating strategy is provided in the Supplementary Information.
